# A Curcumin‐Modified Coordination Polymers with ROS Scavenging and Macrophage Phenotype Regulating Properties for Efficient Ulcerative Colitis Treatment

**DOI:** 10.1002/advs.202300601

**Published:** 2023-05-17

**Authors:** Hang Yao, Feifei Wang, Hui Chong, Jingjing Wang, Yang Bai, Meng Du, Xiaohui Yuan, Xiaofei Yang, Ming Wu, Yuping Li, Huan Pang

**Affiliations:** ^1^ School of Chemistry and Chemical Engineering Yangzhou University Jiangsu 225002 China; ^2^ School of Pharmacy Changzhou University Changzhou Jiangsu 213164 P. R. China; ^3^ State Key Laboratory of Coordination Chemistry Nanjing University Nanjing Jiangsu 210023 P. R. China; ^4^ Department of Neurosurgery Clinical Medical College Yangzhou University Jiangsu 225002 P. R. China

**Keywords:** colitis, nano‐enzyme, oral administration, pH‐control drug delivery, Prussian blue analog

## Abstract

Overexpression of classically activated macrophages (M1) subtypes and assessed reactive oxygen species (ROS) levels are often observed in patients with ulcerative colitis. At present, the treatment system of these two problems has yet to be established. Here, the chemotherapy drug curcumin (CCM) is decorated with Prussian blue analogs in a straightforward and cost‐saving manner. Modified CCM can be released in inflammatory tissue (acidic environment), eventually causing M1 macrophages to transform into M2 macrophages and inhibiting pro‐inflammatory factors. Co(III) and Fe(II) have abundant valence variations, and the lower REDOX potential in CCM‐CoFe PBA enables ROS clearance through multi‐nanomase activity. In addition, CCM‐CoFe PBA effectively alleviated the symptoms of UC mice induced by DSS and inhibited the progression of the disease. Therefore, the present material may be used as a new therapeutic agent for UC.

## Graphical abstract

The chemotherapy drug curcumin (CCM) is decorated with a Prussian blue analog. The modified CCM can be released in the inflammatory tissue (acidic environment), eventually transforming M1 macrophages into M2 macrophages and inhibiting pro‐inflammatory factors. Co(III) and Fe(II) have abundant valence variations, and the low REDOX potential of CCM‐CoFe PBA can remove ROS through multi‐nanomase activity.

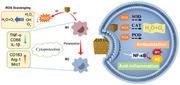

## 1 Introduction

An incapacitating, non‐infectious, and incurable immune‐mediated inflammatory disease is inflammatory bowel disease (IBD), which generally caused widespread health concerns.^[^ ^1^ ^]^ Currently, ulcerative colitis (UC) was regarded as one of the most common clinical phenotypes of IBD.^[^ ^2^ ^]^ Although various anti‐inflammatory chemotherapy drugs have been used in different stages of UC treatment for decades, there are obvious side effects after long‐term use in patients.^[^ ^3^ ^]^ In addition, a large dose was required partially due to the low solubility of drug (curcumin, for instance), and obvious side effects emerged upon long‐term application in UC patients.^[^ ^4^ ^]^ Development of innovative therapeutic approaches with enhanced impact against UC is therefore urgently needed.

Macrophages played a critical role in maintaining the balance of physiological immune environment.^[^ ^5^ ^]^ With certain stimulation, interconversion of M1 and M2 subtypes could be achieved.^[^ ^6^ ^]^ For example, it can be used to treat sepsis and other chronic diseases.^[^ ^7^, ^8^ ^]^ ROS was one of the factors that could regulate the transformation of M1/M2.^[^ ^9^ ^]^ Therefore, the inducement of M1 to M2 subtype transformation highly favored the cure of UC. For instance, Hou et al. synthesized Ti_3_C_2_ NSs which showed good stability under acidic conditions by the two‐step stripping method.^[^ ^10^ ^]^ The synthetic Ti_3_C_2_ NSs can significantly inhibit the inflammatory response by reducing the secretion of pro‐inflammatory factors, increasing the infiltration of M2‐phenotype macrophages, and secretion of anti‐inflammatory factors, so as to effectively reduce the symptoms of colitis. So far, there have been a few articles applying macrophage conversion and ROS downregulation treatment strategies in the treatment of rheumatoid arthritis and arteriosclerosis,^[^ ^11^, ^12^, ^13^ ^]^ but there is almost no research on the treatment of colitis.

Many inflammatory diseases are driven by the production of ROS, including superoxide anions (•O_2_^−^) and hydroxyl groups (•OH), as well as non‐free radicals such as singlet oxygen (^1^O_2_) and hydrogen peroxide (H_2_O_2_). Excess ROS can lead to lipid peroxidation, protein oxidation, and DNA damage, so removing ROS is a top priority.^[^ ^14^ ^]^ Inspired by biology, Zhu et al. developed a novel ROS scavenging sutures for wound treatment by covering surgical sutures with galacylated acid (GA) ‐based nanoparticles (GANPs). The resulting GANPS‐coated sutures can effectively promote wound closure by maintaining tension and reducing ROS levels around the wound.^[^ ^15^ ^]^

Metal–Organic Frameworks (MOFs) are currently one of the attractive materials that displayed attractive properties and potential application in various fields, including drug‐nano‐carriers.^[^ ^16^ ^]^ Owing to the combination of metal centers and functional organic ligands, materials with potent performance of targeting, drug delivery, and ROS regulation have been constructed.^[^ ^17^ ^]^ Yet, little concern about MOFs as biomaterial has arised in the aspect of stability.^[^ ^18^ ^]^ Resembling that of MOFs, coordination polymer (CPs) is a kind of porous material with great effect.^[^ ^19^ ^]^ Due to its characteristics of adjustable chemical composition, high porosity, and large specific surface area, CPs has attracted wide attention. CPs exhibited relatively acceptable stability as biomaterials.^[^ ^20^ ^]^ Prussian blue analogs (PBAs) are typical porous coordination polymer materials and can generally be described by the formula of A_x_M_a_[M_b_(CN)_6_]_y_·nH_2_O, where A is an alkali metal ion, such as Na^+^ or K^+^, and M_a_ and M_b_ represent transition metal cations.^[^ ^21^ ^]^ In this material, the carbon coordination transition metal cations and nitrogen coordination transition metal cations are bridged by cyanide groups respectively, thus forming an open framework.^[^ ^22^ ^]^ In recent years, PBAs have effectively simulated the function of ROS scavenging enzymes, thus showing promising applications in biotechnology.

**Figure 1:**
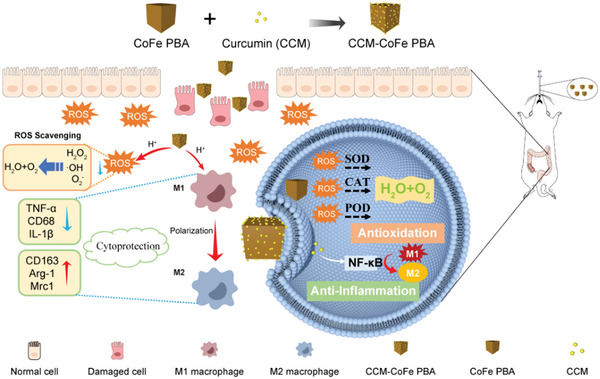
Schematic diagram of oral CCM‐CoFe PBA for the treatment of UC in mice.

In this manuscript, we decorated the chemo drug CCM on Prussian Blue analog through straight‐forward protocol. The synthesized nanohybrid (CCM‐CoFe PBA) exhibited promising biocompatibility. Co(III) and Fe(II) had plentiful changing valence states and a low REDOX potential in CCM‐CoFe PBA enabled the activity to scavenge ROS such as •OH and H_2_O_2_ through multi‐enzyme activity. The decorated CCM could be released in an acidic tissue environment (pH < 7.0), which eventually led to the formation of M2 macrophage and secretion of inhibitory proinflammatory factor. In vivo experiment in DSS‐induced UC mice showed the nanohybrid could effectively alleviate symptoms and inhibited the progress of the disease. Thus, the current material could be potentially applied as novel therapeutic agent toward UC (**Figure** **1**).

## 2 Results and Discussion

### 2.1 Preparation and Characterization of CoFe PBA and CCM‐CoFe PBA

**Figure 2:**
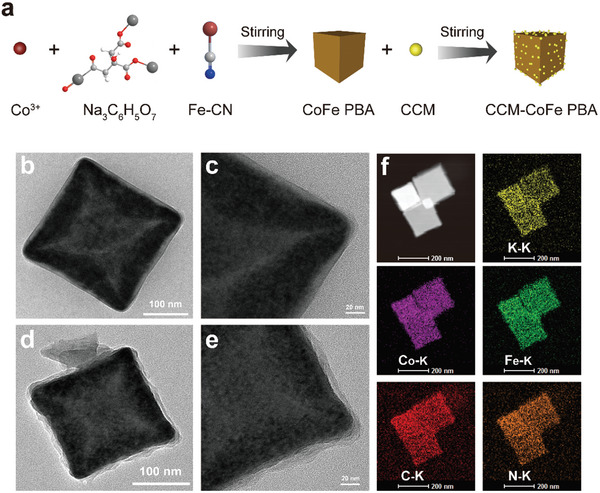
a) Schematic diagram of CCM‐CoFe PBA synthesis process. Transmission electron microscope images of b,c) CoFe PBA, d,e) CCM‐CoFe PBA. f) EDX element mapping image of CCM‐CoFe PBA.

As depicted in **Figure** **2**a, CoFe PBA was synthesized in a routine way by reacting Co(NO_3_)_2_•6H_2_O, citrate sodium and K_3_[Fe(CN)_6_] in ddH_2_O. Subsequently, the resulting CoFe PBA was further dispersed in absolute ethanol in the presence of CCM to yield desired CCM‐CoFe PBA. CoFe PBA and CCM‐CoFe PBA were uniformly cubic particles with average length of ≈200 nm as characterized by TEM (Figure 2b–e) and SEM (Figure S1, Supporting Information). TEM images showed CoFe PBA had a relatively smooth surface, whereas CCM‐CoFe PBA had a core‐shell structure (Figure 2d,e). The opaque shell might be CCM absorbed on the surface of CoFe PBA. The hydrodynamic diameter of CoFe PBA and CCM‐CoFe PBA was measured to be around 400 nm by dynamic light scattering (Figure S2, Supporting Information). The diameter of both cubic materials was calculated to be around 346 nm, therefore, the size data from DLS agreed with the results of TEM and SEM. Furthermore, the average size of the nanohybrid may facilitate CCM‐CoFe PBA's ability to target inflamed colons from the endothelium.^[^ ^23^ ^]^

**Figure 3:**
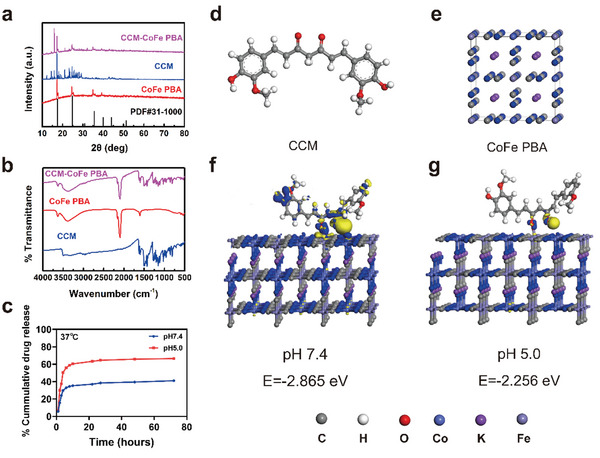
Differences in charge densities of geometric configurations of curcumin interacting with the Fe site on CoFe PBA at different pH states: a) XRD patterns and b) FT‐IR spectra of samples. c) Drug release curves of CCM‐CoFe and PBA at different pH values. d) structure of curcumin. e) Structure of CoFe PBA. f) Adsorption energy of curcumin on CoFe PBA at pH 7.4 and g) adsorption energy of curcumin on CoFe PBA at pH 5.0.

**Figure 4:**
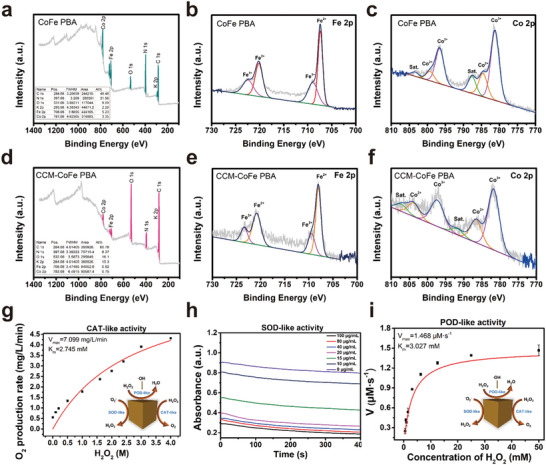
Nanomase activity of CoFe PBA. a‐f) The high‐resolution XPS profiles of a) CoFe PBA survey, b) CoFe PBA Fe 2P, c) CoFe PBA Co 2P, d) CCM‐CoFe PBA survey, e) CCM‐CoFe PBA Fe 2P, f) CCM‐CoFe PBA Co 2P. g) Steady‐state kinetic determination of catalase (CAT) ‐like activity of CoFe PBA with different concentrations of hydrogen peroxide. h) Effects of different CoFe PBA concentrations on superoxide ion elimination: CoFe PBA (0‐100 µg mL^−1^), riboflavin (1.2 mm), methionine (0.13 m), NBT (1 mg mL^−1^) and EDTA∙Na_2_ (0.1 m), pH 7.4. i) Steady‐state kinetic determination of peroxidase (POD) ‐like activity of CoFe PBA with different hydrogen peroxide concentrations (0.3–60 mm).

The EDX elemental mapping image reveals that the four elements C, N, K, Co, and Fe coexist and distribute uniformly (Figure 2f). As shown in **Figure** **3**a, the X‐ray diffraction (XRD) signals of CoFe PBA emerged at 2*θ* values of 17.58, 24.92, 30.91, 35.59, 40.04, 44.14, and 51.28°, which was identical with the characteristic peaks of K_3_CoFe(CN)_6_ (standard card of JCPDS card no. 46–0907).^[^ ^24^ ^]^ The XRD pattern of CCM‐CoFe PBA showed both signals of CCM and CoFe PBA, indicating the existence of CCM in synthesized hybrid material (Figure 3a). The FTIR spectra of CCM, CoFe PBA, and CCM‐CoFe PBA were recorded. As shown in Figure 3b, the strong signal of CoFe PBA at 2105 cm^−1^ could be assigned to cyano anions. In the case of curcumin, the band at 3490 cm^−1^ could be assigned to the vibration of both free bulk hydroxyl group in the phenolics and the intramolecular hydrogen bond in the enol group. The rest peaks that appeared at 1511, 1279, and 1152 cm^−1^ were attributed to the stretch of C=C, aromatic C—O, and C—O—C of the benzene ring and molecular skeleton, respectively. In the case of CCM‐CoFe PBA, characteristic signal of CoFe PBA and CCM appeared in the spectrum of CCM CoFe PBA. To note, the tensile peak of the CCM shifted from 3490 to 3426 cm^−1^, indicating the possible binding site between CCM and CoFe PBA. Typical Raman signals of CoFe PBA (≈500 and 2100 cm^−1^) disappeared in final hybrid material (Figure S3, Supporting Information), this also successfully suggested the absorption of CCM. The *ζ* potentials of CoFe PBA and CCM‐CoFe PBA were measured to be −29.9 and −28.6 mV, respectively (Table S1, Supporting Information), indicating the binding did not play a significant role in changing electronic property of the metal core. The X‐ray photoelectron spectroscopy (XPS) data showed characteristic peaks of Co 2p, Fe 2p, O 1s, N 1s, and C 1s in both CoFe PBA and CCM‐CoFe PBA (**Figure** **4**a–f; Figure S4a–h, Supporting Information). The binding energies of N 1s, O 1s, and K 2p of CCM‐CoFe PBA increased by around 1 eV in contrast to CoFe PBA, indicating the potential influence of electronic structures of CCM decoration. In addition, a new C 1s signal emerged centered at 286 eV, which should be assigned to the decorated CCM molecule.

### 2.2 Acid‐Assisted Release of CCM

There are two potential binding sites of CCM toward CoFe PBA, which were phenolic and enol hydroxyls, respectively. In order to elaborate detailed interaction patterns between CCM and CoFe PBA, we conducted density functional theory calculation (DFT). The binding site was predicted to be the enol segment rather than phenolic hydroxyls from CCM and Fe^3+^ from CoFe PBA, this was suspected to be the reason for limited influence of surface electronic property of CCM binding. Considering the acidic environment in inflamed tissue, we further calculated the binding energy of CCM on CoFe PBA. The results showed weaker interaction and reduced binding energy between CCM and CoFe PBA at acidic (pH 5.0, binding energy of −2.256 eV) condition compared to physiological (pH 7.4, binding energy of −2.865 eV) conditions (Figure 3f,g). The reason for relatively weaker binding of CCM in acidic environment was suspected to be acid assisted break of coordination bond between the Fe^3+^ in CoFe PBA and the keto‐type structure.

It was well documented that inflamed tissue was acidic in nature.^[^ ^25^ ^]^ Considering the relatively weak binding of CCM toward CoFe PBA in acidic environment, we monitored the drug release profile of CCM‐CoFe PBA under in vitro acidic condition (pH 5.0) using the absorption value at 425 nm. First, the CCM loading efficiency was determined to be 65.63% based on its molar extinction coefficient. The absorbance at 425 nm increased in a burst manner within the first 24 h and reached the plateau region in the following 48 h. In summary, 63% of CCM was cumulative released in the acidic medium within 72 h, the release effect in the acidic medium was better than that in neutral medium, as shown in Figure 3c.

Furthermore, the colloidal stability of synthesized nanoparticles in multiple biological buffers has been characterized in terms of particle size and zeta potential. In detail, the particle sizes were found to be almost unchanged in testing medium, including H_2_O, 1 × PBS, artificial gastric juice (pH 1.20), stimulated intestinal fluid (pH 6.8 and 7.4) and a mixture of 1 × PBS with 10% fetal bovine serum during the time span of 0–24 h. This suggested the synthesized nanoparticles were stable within the experimental time duration. Yet, the sizes were found to be different in these medium (Figure S5, Supporting Information). In accordance with particle size, the Zeta potentials of particles remained unchanged during 0–24 h time span. To note, the acidic environment (artificial gastric juice) caused positive potential (Table S5, Supporting Information), which might suggest the release of attached CCM.

Combined with the changes in the inflammatory microenvironment, the synthesized polymeric materials with nanomase responsiveness showed good pH sensitivity. In the non‐inflammatory region, the structure of CCM‐CoFe PBA was stable and the drug release was small. When the CCM‐CoFe PBA reaches the inflammatory zone, the acid sensitivity of CCM‐CoFe PBA will destroy its structure when the pH of the environment becomes weakly acidic, and then the CCM will be released into the inflammatory zone, so as to achieve the purpose of targeted drug delivery and increase the retention time of nanoparticles in the inflammatory zone.

### 2.3 ROS Scavenging Ability of CoFe PBA In Vitro

High‐resolution XPS spectra revealed the existence of both Fe^3+^/Fe^2+^ and Co^3+^/Co^2+^ redox couples. In detail, high‐resolution Fe 2p spectra (**Figure** **4**b,c), the binding energies of 708.4 and 721.3 eV could be vested in Fe 2p3/2 and Fe 2p1/2 of Fe^2+^, separately. And the binding energies of 709.8 and 723.5 eV could be vested in Fe 2p3/2 and Fe 2p1/2 of Fe^3+^, respectively. Likewise, in the high‐resolution Co 2P spectra (Figure 4e,f), the binding energies of 785.6 and 800.3 eV correspond to Co 2P1/2 and Co 2P1/2 of Co^2+^, and the binding energies of 782.1 and 797.4 eV correspond to Co 2P1/2 and Co 2P1/2 of Co^3+^, respectively. Both Co^3+^/Co^2+^ and Fe^3+^/Fe^2+^ REDOX couples might provide abundant REDOX reactions sites for reactive oxygen species elimination.^[^ ^26^ ^]^ H_2_O_2_ and O_2_^−^ were two representative ROS that were extensively produced in various cellular metabolic processes. They were both highly destructive once their amounts exceed the cellular antioxidant capacity by reacting with bio‐active molecules.^[^ ^27^ ^]^ So scavenging excess ROS was a strategy to mitigate the colitis response.^[^ ^28^ ^]^ Based on the redox pairs in CoFe PBA, this nanohybrid might play the role of nanoenzymes to eliminate ROS. To understand the ROS eliminating capability of CoFe PBA, solutions containing hydrogen peroxide or O_2_^−^ were freshly prepared and then incubated with CoFe PBA dispersion.

#### 2.3.1 CAT‐Like Enzyme Activity of CoFe PBA In Vitro

H_2_O_2_ was a downstream product of the •O_2_^−^ and displayed resembling toxicity toward biomolecules.^[^ ^29^ ^]^ Catalase (CAT) could catalyze the decomposition of H_2_O_2_ and correspondingly produce water and O_2_.^[^ ^30^ ^]^ Considering the potential application in ulcerative colitis treatment,^[^ ^31^ ^]^ the CAT‐like nanoenzyme capability evaluation was conducted in a weakly acidic environment (pH 5.5). As shown in Figure S6a (Supporting Information), O_2_ was produced upon treatment of H_2_O_2_ by CoFe PBA, and the O_2_ production rate was in a H_2_O_2_ concentration dependent manner within the first 400 s. Upon increasing the concentration of CoFe PBA, the dissolved oxygen gradually increases (Figure S6b, Supporting Information). The CAT enzyme activity of CoFe PBA abided by the Michaelis‐Menten reaction pattern, and the calculated Michaelis constant (*K*_m_) and maximum initial velocity (*V*_max_) were 2.745 mm and 7.099 mg L^−1^ min^−1^, respectively (Figure 4g). When the time reached 10 min, O_2_ generation concentration in physiological environment (pH 7.4) was ≈9 mg L^−1^, in contrary, this concentration amounted to ≈20 mg mL^−1^ in acidic environment (Figure S9, Supporting Information). This might suggest a better H_2_O_2_ decomposition performance in inflammatory tissues.

#### 2.3.2 POD‐Like Enzyme Activity of CoFe PBA In Vitro

Peroxidase (POD) was another enzyme in charge of H_2_O_2_ or •OH decomposition and the product was H_2_O.^[^ ^32^ ^]^ CoFe PBA could oxidize colorless TMB (3,5,3, 5‐tetramethyl benzylamine) to blue oxTMB in the presence of H_2_O_2_ (absorption maxima at 625 nm). This indicated that the conversion H_2_O_2_ or •OH into non‐toxic and harmless water through the POD‐like enzyme activity of CoFe PBA. The *K*_m_ values of CoFe PBA against H_2_O_2_ and TMB were determined to be 3.027 and 2.092 mm, respectively. The corresponding *V*_max_ values for both substrates were 1.468 and 0.887 µm s^−1^, respectively (Figure 4i; Figure S7b, Supporting Information). Therefore, CoFe PBA has enzyme‐like peroxide activity and also has enzyme‐like oxidation activity. Moreover, it was found that the activity of POD‐like enzymes increased with the increase of CoFe PBA concentration (Figure S7, Supporting Information). The redox properties of both Co^3+^/Co^2+^ and Fe^3+^/Fe^2+^ could be suspected to be the reason for both nanoenzyme activity.

#### 2.3.3 SOD‐Like Enzyme Activity Test of CoFe PBA In Vitro

The superoxide dismutase (SOD) enables the transformation of •O_2_^−^ to H_2_O_2_.^[^ ^33^ ^]^ Oxidation/reduction cycles were involved in this process; therefore, we tested the SOD‐like nanoenzyme activity of CoFe‐PBA. As shown in Figure S8 (Supporting Information), the absorbance of Nitrotetrazolium chloride (NBT, •O_2_^−^ sensor) reduced with increasing concentration of CoFe PBA (5–100 µg mL^−1^). This indicated the scavenging of •O_2_^−^ was in a CoFe PBA concentration dependent manner.^[^ ^34^ ^]^ In addition, the absorbance of NBT at 560 nm was regarded as the pointer for •O_2_^−^ scavenging activity.^[^ ^35^ ^]^ As you saw in Figure 4h, the absorption at 560 nm of NBT upon treatment with 100 µg mL^−1^ CoFe PBA for 400 s in physiological environment, indicating a promising •O_2_^−^ scavenging activity. Therefore, CoFe PBA had good SOD mimicking activity and could catalyze the formation of H_2_O_2_ and O_2_ from •O_2_^−^. We further recorded the XPS spectra of CoFe‐PBA and CCM‐CoFe‐PBA after nanoenzyme catalysis. The results indicated slightly reduce of both Co and Fe peaks. This might suggest the two metal centers played a critical role in the nanoenzyme catalysis (Figure S10, Supporting Information). Taken together, the incorporation of Co ions enhanced the nanoenzyme effect of the original material. CoFe PBA has three kinds of nanoenzyme activities at the same time, including POD, CAT, and SOD, which can effectively inhibit or reduce oxidative damage caused by ROS in vivo.

### 2.4 Detection of Intracellular ROS Clearance

Based on the promising activity of CoFe PBA on ROS scavenging in vitro, we further examined the corresponding performance at the cellular level.^[^ ^36^ ^]^ H_2_O_2_ and lipopolysaccharide (LPS) could increase the inflammatory response of macrophages.^[^ ^37^ ^]^ The mouse macrophage RAW264.7 cells were pretreated with LPS, which could increase ROS production. As shown in Figure S11b (Supporting Information), incubation with LPS lighted up the fluorescent ROS sensor 2′,7′‐dichlorofluorescein diacetate (DCFH‐DA), indicating generation of ROS.^[^ ^38^ ^]^ Subsequently, the LPS‐treated cells were incubated with CoFe‐PBA and CCM‐CoFe‐PBA at the concentration of 3 and 5 mg L^−1^, respectively. Compared to control and LPS treated groups, the fluorescent intensity obviously reduced in a nanoenzyme concentration dependent manner (Figure S11, Supporting Information). This suggested the nanoenzyme could remove total intracellular ROS in vivo and protect RAW264.7 cells from oxidative damage.

### 2.5 Cytotoxicity Assay

The cytotoxicity of CCM, CoFe PBA, and CCM‐CoFe PBA was analyzed in mouse fibroblast L929 and macrophage RAW264.7 cell lines by conventional CCK‐8 assay. CCM showed no obvious 24 h cytotoxicity (≈100% cell survival) within concentration of 1–8 µmol L^−1^ in both cell lines (Figure S12a, Supporting Information). CoFe PBA displayed neglectable cell toxicity in the concentration range of 1–10 mg L^−1^ in L929 cell line, whereas the corresponding cytotoxicity in RAW264.7 cell line was ≈25%. To note, CCM‐CoFe‐PBA displayed neglectable 24 h cytotoxicity (≈100% survival rate) within the concentration range of 1–5 mg L^−1^ in L929 and RAW264.7 cell line (Figure S12c, Supporting Information). Calcein acetoxymethyl ester (calcein‐AM) and propidium iodide (PI) staining method showed singly green but no red fluorescence upon the treatments of three substances in identical condition in both cell lines (Figure S12d, Supporting Information). The result agreed with that of CCK‐8 assay. These results indicate that CoFe PBA and CCM‐CoFe PBA had acceptable biocompatibility and potentially ensured the subsequently biomedical applications.

### 2.6 CCM‐CoFe PBA Induces an Anti‐Inflammatory Response in Macrophages

**Figure 5:**
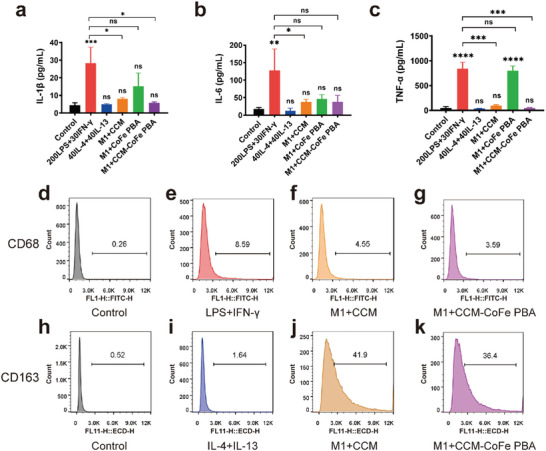
Anti‐inflammatory response of macrophages induced by CCM‐CoFe PBA, CoFe PBA, and CCM in vitro. a–c) The effects of CCM‐CoFe PBA, CCM and CoFe PBA treatment on the expression of IL‐1*β*, IL‐6 and TNF‐*α* were quantitatively detected by ELISA. d–k) Flow cytometry was used to investigate the effect of CCM‐CoFe PBA and CCM on the polarization of mouse macrophage RAW264.7 into M2 type after LPS+IFN‐*γ* activation into M1 phenotype. The results are expressed as means ± SD (standard deviation) of triplicate experiments. A one‐way ANOVA test of multiple comparisons followed by Dunnett's post‐hoc test was used in all analyses. * *p* < 0.05, ^**^ *p* < 0.01, ^***^ *p* < 0.001, ^****^ *p* < 0.0001, ns, not significant.

Among inflammatory cells, M1 macrophage formation responds to various biological signals, especially excess ROS.^[^ ^39^ ^]^ In ELISA experiments (**Figure** **5**a–c), LPS and IFN‐*γ*‐stimulated RAW264.7 significantly increased the expression of typical pro‐inflammatory cytokines (TNF‐*α*, IL‐6, and IL‐1*β*). Subsequently, treatment with CCM, CoFe PBA, and CCM‐CoFe PBA in M1 phenotype of RAW264.7 cells resulted in different impacts. In detail, CCM and CCM‐CoFe PBA caused reduced expression of all three cytokines to similar level of control group. CoFe PBA caused acceptable reduced expression of IL‐6 but relatively comprised reduction of IL‐1*β* and practically no reduction of TNF‐*α* (Figure 5a–c). This suggested decoration of CCM in the nanohybrid played a significant role in potential therapeutic effect. PBA may play a synergistic effect in scavenging ROS, inhibiting cytokines, and relieving inflammation. Meanwhile, to elucidate the anti‐inflammatory mechanism of CCM‐CoFe PBA, we used real‐time fluorescent quantitative PCR (qRT‐PCR) to detect the relative mRNA expression of M2‐related genes. However, CCM‐CoFe PBA significantly increased Mrc1. This trend was similar to that of IL‐4 and IL‐13‐induced cells, suggesting that CCM‐CoFe PBA exerts an anti‐inflammatory effect by inducing M2 differentiation. In contrast, CoFe PBA alone did not trigger different phenotypic reprogramming of macrophages because it did not drive the anti‐inflammatory response (Figure S13, Supporting Information).

According to reports, M1 macrophages induce pro‐inflammatory polarization by reducing intracellular ROS levels, leading to a shift toward their anti‐inflammatory M2 phenotype, ultimately facilitating colitis therapy and other anti‐inflammatory treatments.^[^ ^40^ ^]^ To understand the effect of CCM‐CoFe PBA on macrophage phenotype, M0 macrophages were initially induced to become M1 macrophages by treatment with LPS and interferon (IFN‐*γ*).^[^ ^41^ ^]^ Subsequently, CCM, CoFe PBA, CCM‐CoFe PBA, and M1 macrophages were incubated for 24 h. Next, macrophage polarization (M1 to M2) was assessed by flow cytometry of induced murine macrophages (Figure 5d–k). Both treatments of CCM and CCM‐CoFe PBA led to decreased expression level of M1 macrophage marker CD68 (≈47.30% and ≈58.21%) compared to the LPS and IFN‐*γ* stimulated group, respectively. Both CCM and CCM‐CoFe PBA significantly increased the expression of M2 macrophage marker CD163. However, no significant expression of CD163 was observed when RAW264.7 cells were treated by combination of IL‐4 and IL‐13 for 24 h, which might be due to insufficient induction time. This shows that CCM‐CoFe PBA removes superoxide, not only reduces the number of M1 macrophages, but also increases the anti‐inflammatory level of M2 macrophages during inflammation. These results suggested that CCM‐CoFe PBA not only inhibited classical M1 activation but also induced M2 polarization through a paracrine mechanism.^[^ ^42^ ^]^

### 2.7 In Vivo Anti‐Inflammatory

The most commonly used animal model of IBD is dextran sodium sulfate DSS‐induced colitis.^[^ ^43^ ^]^ Oral DSS will damage the epithelial cells through drinking, thereby destroying the intestinal barrier, leading to the subsequent invasion of the microbiota in the lumen, and finally triggering inflammation.^[^ ^44^ ^]^ Thus, proinflammatory cytokines and oxidative stress play important roles in colitis.^[^ ^45^ ^]^ Since CCM‐CoFe PBA could inhibit the cellular inflammation induced by LPS, we further evaluated the effect of CCM‐CoFe PBA in the treatment of acute colitis.

**Figure 6:**
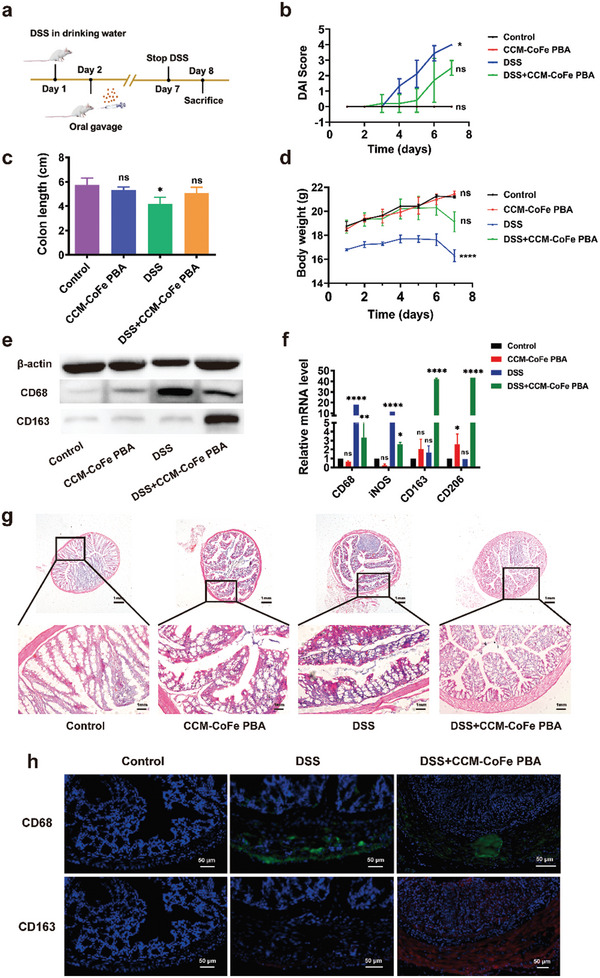
Anti‐inflammatory effect of CCM‐CoFe PBA on DSS‐induced colitis in mice (*n* = 3). a) Schematic of animal experiments. b) Disease activity index (DAI) score. c) Colon length. d) Daily weight change. e) To evaluate the protein expression of M1 (CD68) and M2 (CD163) macrophage markers in colon of mice under different conditions by Western blot analysis. f) Expression levels of CD68, iNOS, CD163 and CD206 in colon tissues of mice. g) Histological section of colon (H&E magnification), the scales are 1 mm. h) Immunofluorescence analysis of type 1 macrophage (CD68 green and 4′,6‐diamidino‐2‐phenylindole [DAPI] blue) and type 2 macrophage (CD163 red and DAPI blue) in colon tissue visualized under confocal microscopy’ scale bar is 50 µm. The results are expressed as means ± SD (standard deviation) of triplicate experiments. A one‐way ANOVA test of multiple comparisons followed by Dunnett's post‐hoc test was used in all analyses. * *p* < 0.05, ^**^ *p* < 0.01, ^****^ *p* < 0.0001, ns, not significant.

Approved by the Laboratory Animal Welfare Ethics Committee of the Yangzhou University, all animal handling procedures were carried out in accordance with the Guidelines for the Care and Use of Experimental Animals of Yangzhou University. Including the control group, all mice were randomly divided into four groups. CCM‐CoFe PBA group, DSS group, and DSS+CCM‐CoFe PBA group. The first two groups gave the mice normal saline, and the remaining group received 3% W/V DSS water. The DSS group and the DSS+CCM‐CoFe PBA group were fed with DSS (3% water solution) for seven successive days to induce UC. The DSS+CCM‐CoFe PBA group received 10 mg Kg^−1^ CCM‐CoFe PBA by gavage every day from the second day, and the other groups received the same amount of normal saline every day from the second day to ensure that all mice received consistent external stimulation (**Figure** **6**a). Multiple parameters including body weight, colon length and bleeding, disease activity index (DAI), histological evidence of inflammation, and expression levels of inflammatory cytokines were assessed.

As demonstrated in Figure 6d, treatment of CCM‐CoFe PBA did not cause significant toxicity in terms of weight loss within the experimental duration. Whereas the administration of singly DSS obviously caused body weight loss. Mice weight gained within the first 5 days and slightly dropped in days 6 to 8 with combinational administration of CCM‐CoFe PBA and DSS. This partially suggested effectively therapeutic effect and good biocompatibility of CCM‐CoFe PBA. Furthermore, administration of CCM‐CoFe PBA did not affect total platelet count, hemoglobin count, white blood cell count or red blood cell count (Figure S14, Supporting Information). Alanine aminotransferase (ALT), aspartate aminotransferase (AST) and blood urea nitrogen (BUN) activities did not change significantly, indicating that CCM‐CoFe PBA had no toxicity to mouse liver and kidney. Histopathological analysis was performed on heart, liver, spleen, lung, kidney, and colon. Hematoxylin and eosin (H&E) staining further indicated that repeated administration of 10 mg kg^−1^ dose of CCM‐CoFe PBA did not cause pathological changes compared to healthy control mice (Figure S15, Supporting Information). We found that even the highest CCM‐CoFe PBA concentration of 10 mg kg^−1^ did not pose a safety risk in vivo.

The in vivo pharmacokinetics of CCM‐CoFe PBA compared to free CCM has been investigated. CCM started to accumulate in kidney, feces, and blood was observed after oral administration of CCM‐CoFe PBA and CCM, respectively. The exact time for peak concentration remained to be determined, but it can be clearly seen that then concentration was relatively high ≈4 h. The concentration gradually dropped to the initial level when time amounted to 24 h. In addition, free CCM and CCM‐CoFe PBA displayed similar biodistribution after 24 h of gavage administration. The CCM component was widely distributed in heart, liver, spleen, kidney, colon, blood, and feces (Figures S18 and S19, Supporting Information). This indicated attaching CCM to nanohybrid did not alter it's in vivo pharmacokinetics. Apart from CCM, we also investigated the in vivo clearance of the nanoparticles using Co ion as the candidate. The concentration of Co ion started to accumulate within 8 h after intragastric administration, and dropped in the time span of 8–24 h. Most concentrated Co ion was found in feces (>100 ng mL^−1^) and the concentration of Co ion in liver, kidney, and blood was below 30 ng mL^−1^. The biodistribution of Co ions after 24 h of intragastric administration of CCM‐CoFe PBA displayed a similar profile compared to the time‐dependent Co ions distribution (Figures S20 and S21, Supporting Information). This suggested a quick in vivo clearance.

It is known that DAI is an indicator comprehensively evaluated based on changes in body weight, consistency of stool, and rectal bleeding (Figure 6b). Compared with the control group, the DSS group, which is mainly characterized by bloody diarrhea, sustained weight loss (Figure 6d), and high mortality, developed severe colitis and significantly increased DAI levels. Mice in the DSS+CCM‐CoFe PBA group with 10 mg kg^−1^ had slower weight loss, significantly improved survival rate, and significantly decreased DAI level. Although the mice in the DSS+CCM‐CoFe PBA group could not reach the same level as the normal control group (≈0.5 cm shorter) but obviously longer than that of DSS treatment group, the results showed that the mice had an acceptable therapeutic effect. The colons of mice after 7 days of DSS induction showed obvious symptoms of congestion, inflammation, hematochezia, diarrhea, and shorter length. After CCM‐CoFe PBA treatment, the colon of mice showed no obvious signs of macroscopic inflammation, the hematochezia and diarrhea were improved, and the length of the colon became longer still shorter than that of the control group (Figure 6c; Figure S16, Supporting Information). This indirectly suggested that DSS‐induced colitis was partially relieved.

Macrophages play an important role in the pathophysiology of ulcerative colitis.^[^ ^46^ ^]^ In DSS‐induced colitis mice, Ulcerative colitis is characterized by elevated levels of several inflammatory markers.^[^ ^47^ ^]^ The transcriptional results showed upregulation of proinflammatory cytokines (CD86, IL‐1*β*) and induced nitric oxide synthase (iNOS) after administration of DSS compared to the control group (Figure 6f) showed the severity of the disease. The CCM‐CoFe PBA group was fed with 10 mg kg^−1^, which significantly reduced the production of inflammatory cytokines in serum, while the expression of CD206 (M2 macrophage marker) and CD163 (M2 macrophage marker) were upregulated compared to the DSS model group, suggesting the tendency of recovery in signaling path level. Western blotting was used to detect the protein levels of CD68 (M1 marker) and CD163 (M2 marker) in colon tissue. Compared with the DSS‐induced group, the expression patterns of CD68 and CD163 proteins were decreased and increased, respectively, after CCM‐CoFe PBA administration (Figure 6e). This agreed with the result of flow cytometry. The inhibitory effect of CCM‐CoFe PBA on M1 polarization further proved by immunofluorescence experiments, which the area of green fluorescence of CD68 reduced and red fluorescence of CD163 increased after treatment of CCM‐CoFe PBA comparing to the DSS group and control group (Figure 6h). These results showed that the differentiation of M2 macrophages was enhanced and M1 was inhibited in macrophage polarization. Therefore, we hypothesized that CCM‐CoFe PBA could reduce inflammatory mediators by inhibiting the infiltration and proliferation of macrophages at the site of inflammation, thereby inhibiting colitis caused by DSS. Similar tendencies were found in histological analysis as well (Figure 6g). HE staining showed that compared with the control group, the mucosal structure of the colon tissue in the DSS group was irregular, the crypts were lost and necrotic, a large number of immune cells were infiltrated, the goblet cells were damaged, and the colon was severely damaged. There are obvious signs of inflammation, including the epithelium. Yet, oral administration of CCM‐CoFe PBA at 10 mg kg^−1^ significantly ameliorated these symptoms of histological inflammation, as demonstrated by a significantly reduced inflammatory response, only slight damage in the mucosal area of the colon, and a small number of immune cells may infiltrate the mucosa, muscle, or submucosa. Also, the majority of goblet cells were preserved in the crypts, the structure of the intestinal villi was restored, and the colonic thick epithelium was regenerated. These findings demonstrate that the prepared CCM‐CoFe PBA possesses good anti‐inflammatory activity and can effectively relieve colitis induced by DSS in mice.

## 3 Conclusion

In summary, the CoFe PBA was synthesized by a simple and low‐cost strategy and it displayed good biosafety. As a highly efficient ROS scavenger, CoFe PBA produced in this way effectively converted harmful ROS such as •OH, hydrogen peroxide, and •O_2_^−^ into H_2_O and O_2_, alleviating oxidative stress in inflamed tissues. The average size of CoFe PBA is ≈200 nm, and CoFe PBA preferentially accumulates at the site of intestinal mucosal inflammation through a size‐dependent mechanism. The potential anti‐colitis drug CCM was further decorated as synthesized nanohybrid. Due to the synergistic abilities of ROS clearing and proinflammatory cytokines inhibiting, CCM‐CoFe PBA significantly reduced symptoms of colitis without adverse side effects in mice model. This study provides important evidence that CCM‐CoFe PBA protects UC in live animals and provides a potential alternative therapy for UC patients. More importantly, CoFe PBA could be applied as a platform for general inflammation treatment by decoration with accordingly chemical drugs.

## Conflict of Interest

The authors declare no conflict of interest.

## Supporting information

Supporting Information

## Data Availability

Research data are not shared.
