# Supplementary material for: A Curcumin‐Modified Coordination Polymers with ROS Scavenging and Macrophage Phenotype Regulating Properties for Efficient Ulcerative Colitis Treatment
Source: Adv Sci (Weinh). 2023 May 17;10(19):2300601. doi: 10.1002/advs.202300601 (PMC10323614; doi:10.1002/advs.202300601)
Supplement: Supplementary file 1 — Supporting Information [file ADVS-10-2300601-s001.pdf]

ADVANCED SCIENCE
Open Access

Supporting Information

for *Adv. Sci.*, DOI 10.1002/advs.202300601

A Curcumin-Modified Coordination Polymers with ROS Scavenging and Macrophage Phenotype Regulating Properties for Efficient Ulcerative Colitis Treatment

*Hang Yao, Feifei Wang, Hui Chong, Jingjing Wang, Yang Bai, Meng Du, Xiaohui Yuan, Xiaofei Yang, Ming Wu, Yuping Li and Huan Pang**

Supporting Information

A Curcumin-Modified Coordination Polymers with ROS Scavenging and Macrophage Phenotype Regulating Properties for Efficient Ulcerative Colitis Treatment

*Hang Yao[#], Feifei Wang[#], Hui Chong, Jingjing Wang, Yang Bai, Meng Du, Xiaohui Yuan, Xiaofei Yang, Ming Wu, Yuping Li, Huan Pang**

F. Wang, J. Wang, M. Du, X. Yuan, X. Yang, M. Wu, Prof. H. Yao, Prof. H. Chong, Prof. H. Pang
School of Chemistry and Chemical Engineering, Yangzhou University, Jiangsu 225002, China.

Prof. Y. Bai
School of Pharmacy, Changzhou University, Changzhou, Jiangsu, 213164, P. R. China.
State Key Laboratory of Coordination Chemistry, Nanjing University, Nanjing, Jiangsu, 210023, P. R. China.

Prof. Y. Li
Department of Neurosurgery, Clinical Medical College, Yangzhou University, Jiangsu 225002, China.

[#] These authors contributed equally to this work.
E-mail: huanpangchem@hotmail.com; panghuan@yzu.edu.cn

Experimental Section

Materials and instruments

Formalin, acetic acid, H_2O_2, hydrochloric acid, and $K_3[Fe(CN)_6]$ were all provided by HUSHI (Shanghai, China). Sodium hydroxide, EDTA·Na_2, Tween-20, $Co(NO_3)_2·6H_2O$, $NaHCO_3$, and absolute ethanol were all provided by Aladdin (Shanghai, China). TMB (3,3,5, -tetramethylbenzidine) was purchased by Shjsbio (Shanghai, China). Riboflavin, methionine, nitrotetrazolium chloride, and curcumin were purchased by Sangon Biotech (Shanghai, China). DMEM medium, double antibody (PS), and sodium pyruvate solution were provided by Gibco. Fetal bovine serum was purchased by Corning. Lipopolysaccharide (LPS) was

provided by Sigma Aldrich (St. Louis, MO). Interferon-γ (IFN-γ) was provided by Sino Biological. Interleukin-4 (IL-4) and interleukin-13 (IL-13) were provided by Peprotech. Cell lysate (Trizol) was provided by Takara (Japan), and SYBR Green qPCR Mix was purchased by Beyotime (China). Chloroform was provided by Sinopharm (Shanghai, China). Sodium citrate and isopropanol were provided by Macklin (China). Dextran sulfate sodium (DSS) was purchased by MP Biomedical (Santa Ana, CA, USA). DAPI provided by Sigma. OCT cryoembedding agent provided by Leica. RIPA lysates, protease inhibitors, PMSF, nucleases, hematoxylin, and eosin (H&E), SDS-PAGE gel preparation kit were provided by Solar Science & Technology Co., Ltd. (Beijing, China).

The morphology of samples was observed by scanning electron microscope (SEM, Zeiss_Supra55) under the acceleration voltage of 5.0 kV. Transmission electron microscopy (TEM) investigations were performed by a JEM-2100 instrument. The phase and crystal structure of the material was characterized by X-ray diffraction (XRD) on a Bruker D8 Advanced X-ray Diffractometer (Cu-Kα radiation: λ = 0.15406 nm). The chemical states were measured using an Axis Ultra X-ray photoelectron spectroscope (XPS, Thermo Fisher Scientific ESCALAB250Xi) equipped with a standard monochromatic Al-Kα source (HV = 1486.6 eV). Fourier transform infrared (FTIR) transmission spectra were obtained on a BRUKER-EQUINOX-55 IR spectrophotometer. Raman measurement was obtained on Raman Microscopic Imaging Spectrometer (DXRxi). The absorption spectrum of the material was examined on a UV-visible spectrometer (Shimadzu, Japan). The hydrodynamic diameter and Zeta potential of the material were measured on a nanoparticle size analyzer (Malvern Panalytical, England). Gel imager (CLINX), non-contact ultrasonic Cell Pulverizer (Ningbo Xinzhi Biotechnology Co., LTD.), fluorescence quantitative PCR instrument (MyGo Pro), NanoDrop One (Thermo Scientific), PCR instrument (Bio-Rad), Fluorescence inverted microscope (OLYMPUS), microplate reader (Tecan Spark), freezing microtome (LEICA CM1950), Ultraviolet-visible spectrophotometer (Shimadzu, Japan).

Synthesis of CoFe PBA and CCM-CoFe PBA

Dissolve 0.873 g $Co(NO_3)_2 \cdot 6H_2O$ and 1.3234 g sodium citrate in 100 mL of deionized water, and stir until completely dissolved; Meanwhile, dissolve 0.6584 g $K_3[Fe(CN)_6]$ in 100 mL of deionized water. Afterwards, the $K_3[Fe(CN)_6]$ aqueous solution was poured into the aqueous solution of $Co(NO_3)_2 \cdot 6H_2O$ and sodium citrate and stirred for 20 min. CoFe PBA was collected by a centrifuge.

50 mg CoFe PBA was weighed in the bottle and 5 mL absolute ethanol was added, then the lid was put into the ultrasonicator and dispersed evenly. Then 50 mg curcumin was added and mixxed for 24 h. The material was then used deionized water washing and leaving to dry.

Adsorption energy calculation method

The value of adsorption energy can determine whether the reaction can proceed spontaneously. The calculation method of adsorption energy is as follows:

$\Delta E_{ads} = E_{slab/adsorbate} - E_{slab} - E_{adsorbate}$,

Where, ΔE_{ads} is adsorption energy, kcal/mol; Wateractivated on the E_{slab}/ Wateractivated system total energy, kcal/mol; E_{slab} is the total energy of the crystal surface before the reaction, kcal/mol; $E_{waterbate}$ is the energy on the prereaction adsorption, kcal/mol. If the adsorption energy is greater than zero, it cannot proceed spontaneously; if the adsorption energy is less than zero, it can proceed spontaneously. The lower the absolute value of the adsorption energy, the more likely the reaction will occur spontaneously.

Drug loading and drug encapsulation rate

Decompose an appropriate amount of dry CCM-CoFe PBA and dilute to 2 mL of absolute ethanol with 50 μL of hydrochloric acid (2 M). The drug loading and encapsulation efficiency were calculated by the standard curve and calculation formula, and the ultraviolet-visible spectrum analysis was carried out at a wavelength of 425 nm:

DLE (%) = (amount of loaded drug)/(total amount of feeding drug) ×100%

EE (%) = (amount of loaded drug)/(amount of drug loaded NPs) ×100%

Drug release

Dissolve 2 mg of CCM-CoFe PBA in 20 mL of PBS and Tween-20 solution (pH = 5.0), shaking continuously at 37 °C (120 rpm). 1 mL of the solution was centrifuged, and at selected time intervals (0, 1, 2, 3, 5, 18, 36, 48 and 60 hours), replaced with 1 mL of PBS solution. Curcumin release was measured by UV-Vis at 425 nm and combined with a calibration curve. The cumulative percent release was also calculated over the selected time interval.

Colloidal Stability of CCM-CoFe PBA

Add a certain amount of CCM-CoFe PBA to three simulated intestinal fluid environments (pH = 1.2 artificial gastric fluid (ChP), pH = 6.8 artificial intestinal fluid (SIF),

pH = 7.4 SIF), as well as H_2O, PBS, and PBS containing 10% FBS. Prepare a solution of 0.01mg/mL, and evaluate the colloidal stability of CCM-CoFe PBA in different biological buffers by dynamically monitoring particle size changes and Zeta potential changes.

SOD-like enzyme activity measurement

The SOD mimetic activity of CoFe PBA was assessed by measuring the photoreduction inhibition of NBT. In a traditional protocol, riboflavin (1.2 mM), methionine (0.13 M), NBT (1 mg mL^{-1}), EDTA·Na_2 (0.1 M), and CoFe PBA (5-100 μg mL^{-1}) were sequentially added to PBS (pH = 7.4, 0.1 M). The mixed solution was irradiated for 15 min under LED light. Absorbance at 560 nm was measured immediately after illumination ended.

Determination of CAT-like enzyme activity

Determination of CoFe PBA CAT enzyme activity at room temperature by measuring the solubility of O2 produced at different reaction times (in mg L^{-1}) using a specific oxygen electrode on a dissolved oxygen meter (JPBJ-608, Leici, China). Usually, different concentrations of CoFe PBA were mixed with H_2O_2 in 5.0 mL of PBS buffer (0.1 M, pH = 7.4). Oxygen solubility (mg/L) was monitored with a dissolved oxygen meter.

Kinetic analysis was performed at room temperature in 2 mL reaction buffer solution (0.1 M PBS, pH = 7.4) and 2 mL CoFe PBA solution.

1) Different concentrations of hydrogen peroxide: 2 mL of different concentrations of hydrogen peroxide (4, 3, 2.4, 2, 1.6, 1, 0.5, 0.2, 0.1, 0.05, 0.002, 0.001, 0.0005, 0.0002 M) were added to the reaction system containing CoFe PBA (80 μg mL^{-1}, 2 mL). Monitor dissolved oxygen (mg L^{-1}) for 10 minutes with a dissolved oxygen detector.

2) Different CoFe PBA concentrations: 2 mL of different concentrations of CoFe PBA solution (0.12, 0.08, 0.04, 0.03, 0.02, 0.016, 0.01, 0.005, 0.002 g L^{-1}) were added to the reaction system containing hydrogen peroxide (4 mM), 2 mL Oxygen solubility (mg L^{-1}) was monitored within 400 s with a dissolved oxygen meter.

All Michaelis-Menten constants were calculated using the Michaelis-Menten saturation curves from Origin software.

Measurement of POD-like enzyme activity

50 μL CoFe PBA (4 g L^{-1}) was mixed with 100 μL hydrogen peroxide (30%, m/m) and 50 μL TMB (0.12 M) in 2.8 mL sodium acetate buffer (0.1 M, pH = 4.0). After incubation for 3 minutes, the absorbance of the mixture at 652 nm was measured with a UV-Vis (Shimadzu

Corporation, Japan). The POD-like activity of CoFe PBA was determined in the presence of hydrogen peroxide using TMB as substrate. To assess capsule-like activity, record the absorbance of the chromogenic reaction (TMB = 652 nm) within a specific reaction time.

In a typical experiment, the reagents were added in the order of CoFe PBA (final concentration 50 μg mL^{-1}), 50 μL TMB (final concentration 2 mM), and 305 μL into hydrogen peroxide (30%, m/m) in 200 μL buffer solution (0.1 M NaAc, pH = 4.0) .Kinetic analysis was performed at room temperature in 2.8 mL of reaction buffer solution (0.1 M NaAc, pH = 4.0) and 20 μL of CoFe PBA solution (final concentration 50 μg mL^{-1}).

1) Hydrogen peroxide as substrate: Add 50 μL of 0.12 M TMB and 100 μL of hydrogen peroxide at various concentrations (0.3, 0.6, 1.2, 2.4, 4.8, 9.6, 19.2, 30, 38.4, 60 mM). Absorbance was recorded over time at 625 nm.

2)TMB as substrate: Add 305 μL of 30% hydrogen peroxide and 50 μL of TMB with different concentrations (6, 12, 24, 30, 48, 60, 96, 120 mM) into the reaction system containing CoFe PBA, and record the method for measuring the absorbance time at 625 nm.

Cell culture

Mouse RAW264.7 and human epithelial cells L929 (ATCC) were cultured in Dulbecco's Modified Eagle's medium (GIBCO) supplemented with 10% fetal bovine serum (Corning).

Cytotoxicity test

The cell counting kit-8 (CCK-8) assay (Beyotime, China) and calcein acetoxymethyl ester/propidium iodide (Calcein AM/PI) cell viability/cytotoxicity assay kit (Beyotime) to detect the effects of different concentrations (16-1000 μg mL^{-1}) of pure curcumin, CoFe PBA and CCM-CoFe PBA on the growth of RAW264.7 (mouse macrophages) and L929 (human epithelial cells). Co-cultivate cells and material for 3 days, and CCK-8 was detected every 24 hours. After the CCK-8 reagent was incubated with the cells for 2 hours, the absorbance was measured with a microplate reader at 450 nm, so as to detect the cell viability. After 3 days, the calcein AM/PI cell viability/cytotoxicity was detected by a kit for detecting cell viability. Live cells (green) or dead cells (red), observed under a fluorescent microscope after incubation with CalceinAM/PI buffer for 30 min.

Intracellular ROS clearance rate detection

The DCFH-DA fluorescent probe was used to monitor intracellular ROS levels, and the excitation wavelengths were 488 nm and 525 nm, respectively. RAW264.7 cells were

cultured in a 24-well plate at a density of 100,000 cells ml^{-1} per well, 1 mL per well, and incubated at 37 °C for 24 hours. Then, 500 ng mL^{-1} LPS was mixed to induce the production of intracellular ROS for 24 hours. Then, 1 mL of 3 mg L^{-1} CoFe PBA, 5 mg L^{-1} CoFe PBA, and CCM-CoFe PBA were added to the corresponding Wells for 24 h. Wash 1 time with serum-free cell culture medium, stain with DCFH-DA for 30 minutes, and wash 3 times with serum-free cell culture medium. Then, 300 μL of PBS was added to each well and observed under a fluorescent inverted microscope.

RNA isolation and real-time PCR

RAW264.7 was inoculated into a 6-well plate at a seeding density of 100,000 mL^{-1}, 2 mL per well, and incubated for 24 hours. To determine the effect of CCM, CoFe PBA, and CCM-CoFe PBA on proinflammatory cytokines, 200 ng mL^{-1} of LPS and 30 ng mL^{-1} of IFN-γ were added to RAW264.7 cells and incubated for 24 h. Then added CCM (6 μmol L^{-1}), CoFePBA (10 mg L^{-1}), CCM-CoFePBA (4.5 mg L^{-1}), and cultured with RAW264.7 cells for 24 hours. Total RNA was extracted using Trizol reagent (Takara) according to the manufacturer's instructions. Then 200 μL chloroform was added, violently shaken for 15 s, and then left for 5 min. Centrifuge the sample at 12,000 rpm for 15 minutes at 4 °C and transfer the supernatant to a new EP tube. After adding 500 μL of isopropanol to a new EP tube, the mixture was shaken, centrifuged (12000 rpm, 10 min, 4 °C), and the sediment was washed once with 75% ethanol. The concentration and purity of RNA was analyzed by measuring the absorbance at 260 nm and 280 nm on a NanoDropone (ThermoScientific). CDNA was synthesized using PrimeScript_II First Strand CDNA Synthesis Kit (Takara). The primers used were synthesized by bio-synthetics, and their sequence is shown in Table S2. β-actin was used as a control. The conditions of PCR are: maintenance time, 5 minutes at 95 °C; PCR stage 95 °C for 15 s, 60 °C for 15 s, 72 °C for 30 s, a total of 45 cycles. Melting curve stage, 95 °C 15 s, 60 °C 15 s, 95 °C 15 s. Synthetic mRNA was expressed normalized to controls by β-actin mRNA. The expression levels of iNOS, MrC1, and β-actin were detected, and the expression levels of target genes were calculated by $2^{-\triangle\triangle CT}$.

Measurement of cytokines

RAW264.7 was inoculated into a 6-well plate at a seeding density of 100,000 mL^{-1}, 2 mL per well, and incubated for 24 hours. Cells were stimulated with 200 ng mL^{-1} of LPS and 30 ng mL^{-1} of IFN-γ for 24 h and then treated with CCM (6 μmol L^{-1}), CoFe PBA (10 mg L^{-1}), and CCM-CoFe PBA (4.5 mg L^{-1}) for 24 h, respectively. The supernatant was collected, and

the concentrations of IL-1β, IL-6 and TNF-α were measured with corresponding ELISA kits (Invitrogen by Thermo Fisher Scientific).

Flow cytometry analysis

RAW264.7 was inoculated into a 6-well plate at a seeding density of 300,000 mL^{-1}, 2 mL per well, and incubated for 24 hours. Cells were stimulated with 200 ng mL^{-1} of LPS and 30 ng mL^{-1} of IFN-γ for 24 h. Cells were then treated with CCM (6 μmol L^{-1}), CoFe PBA (10 mg L^{-1}), and CCM-CoFe PBA (4.5 mg L^{-1}) for 24 hours, respectively. Cells were then washed twice with PBS, and stained with CD68 antibody (rabbit, Proteintech, 1:1000) and CD163 antibody (rabbit, Proteintech, 1:1000) at 4 °C for 30 minutes. When the incubating is over, the primary antibody was washed twice by centrifugation with PBS. And then incubated with PBS (5%) containing serum for 30 min. Then the fluorescent secondary antibody goat anti-rabbit IgG labeled by Alexa 488 (Abcam, 1:2000) was incubated and diluted with serum-containing PBS (5%) for 30 min at 4 °C. When the incubating is over, the secondary antibody was washed again with PBS, then resuspended with 500 μL PBS, and stored at 4 °C away from light. Flow cytometry analysis was performed using a Beckman Coulter (Life Science, USA) platform. Data analysis and plotting were performed using Flowjo software V10.

Animal experiments

Approved by the Laboratory Animal Welfare Ethics Committee of the Yangzhou University, all animal handling procedures were carried out in accordance with the 《Guidelines for the Care and Use of Experimental Animals of Yangzhou University》. Male Balb/c mice (6-7 weeks old, 18-20 g body weight), 5 mice per cage, were used in this study, housed in a temperature-controlled 22 to 25 degrees Celsius with a standard 12/12-hour light/dark Circulating Specific Pathogen Free (SPF) animal facilities. Mice were randomly divided into four groups (normal control group, CCM-CoFe-PBA drug-only group, DSS-induced colitis group, and DSS-induced CCM-CoFe-PBA-treated colitis group) (Table S2). group. Dextran sulfate-containing sterile drinking water. Aqueous colitis in mice for 7 days. The normal control group and CCM-CoFe PBA pure drug group were fed with sterile drinking water normally. CCM-CoFe PBA pure drug group and DSS-induced CCM-CoFe PBA treatment group were given CCM-CoFe PBA (10 mg/Kg) by gavage every day from the first day, and the other two groups received an equal amount of normal saline daily. On day 8, the mice were all sacrificed under EFE anesthesia. Visual stool consistency, weight change,

and fecal bleeding were assessed daily for 8 days. Disease activity index (DAI) was measured (Table S3), including stool consistency index (0-3), body weight loss index (0-4), and stool bleeding index (0-3). On the 9 th day, the mice were sacrificed, and their entire colons were excised under isoflurane anesthesia. Measure the length of the colon and wash it gently with salt water. For histological analysis a portion of the colon is prepared. The heart, liver, spleen, lung, and kidney were collected for further pathological analysis.

The animal experiment approval number for this research is: DWLL-202202-029. The "Guidelines for the Approval of the Animal Care and Use Professional Committee of Yangzhou University" stipulates that in accordance with the 3R principle of experimental animals, all animal experiments are carried out in accordance with the guidelines. The "Permit for Experimental Animals" was issued by the Science and Technology Department of Jiangsu Province (Jiangsu Science and Technology Department) in 2017-0044. The supervision and inspection of animal experiments is under the responsibility of the Animal Welfare and Ethics Committee

Evaluation for Inflammatory Bowel Disease

The mice were weighed every day during the experiment. DAI was assessed at the end of the intervention based on the three dimensions of weight change, rectal bleeding, and stool consistency. Use a ruler to determine the length of the colon. Histopathological analysis of the colon was performed by hematoxylin and eosin staining.

Quantitative real-time polymerase chain reaction

According to the manufacturer's instructions, total RNA was extracted from the colon tissue (100 mg) of the experimental animal group using Trizol reagent (Takara). Add 1 mL Trizol reagent, and cut the tissue into pieces with scissors. Cut into pieces and blow repeatedly with a 10 mL syringe needle. Then 200 μL chloroform was added, violently shaken for 15 s, and then left for 5 min. Centrifuge the sample at 12,000 rpm for 15 minutes at 4°C and transfer the supernatant to a new EP tube. After adding 500 μL of isopropanol to a new EP tube, the mixture was shaken, centrifuged (12000 rpm, 10 min, 4 °C), and the sediment was washed once with 75% ethanol. The concentration and purity of RNA was analyzed by measuring the absorbance at 260 nm and 280 nm on a NanoDropone (ThermoScientific). CDNA was synthesized using PrimeScript_II First Strand CDNA Synthesis Kit (Takara). The primers used were synthesized by bio-synthetics, and their sequence is shown in Table S2. β-actin was used as a control. The conditions of PCR are:

maintenance time, 5 minutes at 95 °C; PCR stage 95 °C for 15 s, 60 °C for 15 s, 72 °C for 30 s, a total of 45 cycles. Melting curve stage, 95 °C 15 s, 60 °C 15 s, 95 °C 15 s. Synthetic mRNA was expressed normalized to controls by β-actin mRNA. The expression levels of CD68, iNOS, CD163, CD206 and β-actin were detected, and the expression levels of target genes were calculated by $2^{-\triangle\triangle CT}$.

Western blotting

Cut the colon tissue into small pieces and put them into 1.5 mL EP tubes. RIPA lysate mixture was prepared (1 mL of RIPA lysate was added with 5 μL protease inhibitor, 5 μL PMSF, RIPA: PMSF = 100:1, now for use) and 2 μL nuclease; 1 mL of pre-cooled RIPA lysate mixture was added to each 1.5 mL EP tube. Homogenize with a homogenizer at low speed, 30 seconds each time, and ice bath for 1 minute between two homogenizations, and then placed for 5 cycles of ultrasonic lysis (ultrasonic 5 s, stop 5 s) until the tissue was completely lysed. Lysis solution immediately transfer the supernatant to a new centrifuge tube in a pre-cooled centrifuge at 1200 rpm and 4 °C for 10 minutes to denature the protein quantitatively. Protein concentrations were determined using BCA protein Concentration Assay Kit (Beyotime). The denatured proteins were separated on 10% SDS-PAGE gels and transferred to PVDF membranes (BIO-RAD). The PVDF membrane was incubated with CD68 antibody (rabbit, Proteintech, 1:1000) and CD163 antibody (rabbit, Proteintech, 1:1000) at 4°C after blocking the PVDF membrane with 5% skim milk for 2 hours, followed by overnight incubation. After washing with TBST, they were incubated with goat anti-rabbit IgG (Thermo, USA, 1:5000) for 1 hour at room temperature. After washing 3 times with PBST (PBS containing 0.1% Tween-20), positive signals were detected with ECL high-sensitivity chemiluminescence detection kit (Vazyme) according to the manufacturer's instructions, and scanned with an imaging system ChemiScope 6000 chemiluminescence detector.

Pathological evaluation

The colons of the normal control group, CCM-CoFe PBA pure drug group, DSS-induced colitis group, and DSS-induced CCM-CoFe PBA treated colitis group was fixed with 10% formalin, conventionally processed, dried, embedded with OCT cryo-embedding agent, and sections with a thickness of 6 microns were processed with H&E and staining. The stained tissue was examined with a light microscope and photographed.

Immunofluorescence

The colons of the normal control group, DSS-induced colitis group, and DSS-induced CCM-CoFe PBA-treated colitis group were fixed with 10% formalin, conventionally processed, dried, and embedded in the OCT cryo embedding agent. The slices were 6 μm thick. Sections were incubated with 0.25% Triton PBS solution for 25 minutes at room temperature, washed 3 times with PBS for 5 minutes/time. Goat serum prepared in 10% PBS was blocked at room temperature for 30 minutes, and washed 3 times with PBS, 5 minutes each time. Antibody CD68 (rabbit, Proteintech, 1:200) and antibody CD163 (rabbit, Proteintech, 1:200) were diluted in 10% goat serum. The negative control group was incubated with 10% goat serum. Incubated overnight at 4 °C in a humid chamber, then washed with PBS 3 times for 5 min each. Secondary antibodies goat anti-rabbit IgG labeled by Alexa 488 (Abcam, 1:500) and goat anti-rabbit IgG labeled by Alexa568 (Abcam, 1:500) were diluted in PBS. Negative controls were incubated with mixed secondary antibodies, incubated 2 h at 37 °C, and then washed with PBS 3 times for 10 min each. Nuclei were stained with DAPI for 30 minutes and washed 3 times with PBS for 5 minutes each. Fluorescence was observed by fluorescence inverted microscope (Olympus Corporation) after the tablets were sealed with a fluorescent sealing agent.

In vivo biosafety

The normal control group, CCM-CoFe PBA pure drug group, and CCM-CoFe PBA treated colitis group induced by DSS were observed by pathology. Seven days after gavage, blood was drawn for routine serum chemistry and hematology. Tissues such as heart, liver, spleen, lung and kidney were collected to observe the in vivo toxicity of CCM-CoFe PBA in major organs. These tissues were collected, washed with deionized water and fixed in 10% neutral buffered formalin. Routine processing, drying, OCT low temperature tissue fixation, 6 μm sections, H&E staining, inspection, light micrographs.

In vivo pharmacokinetics and biodistribution

Free CCM solution (100 mg/kg) and CCM-CoFe PBA solution (100 mg/kg) were orally administered to mice. At predetermined time points (1, 4, 8, 24 h), blood samples were collected from the orbit and centrifuged for 10 minutes (4 ℃, 5000 rpm) to obtain plasma. And then add sodium citrate buffer (50 μL, pH = 3.0) to 150 μL plasma, incubate for 3 min, then add 1.5 mL of methanol and gently rotate to dilute. Centrifuge (4 ℃, 10000 rpm, 10 min)

to obtain the supernatant, and measure the CCM content using a microplate reader under excitation/emission at 420/540 nm.

To measure its biological distribution, we sacrificed mice 24 h after oral administration of free CCM and CCM-CoFe PBA by gavage, and collected their main organs, blood, and feces. After washing and weighing, homogenize the sample and then add sodium citrate buffer (500 μL. pH = 3.0). Rotate with 1 mL of methanol for 10 min. After centrifugation to obtain the supernatant, the CCM concentration in each tissue was determined using the above method.

We conducted *in vivo* pharmacokinetic and biological distribution experiments on the biodegradability of nanoparticles and their clearance ability *in vivo*. CCM-CoFe PBA solution was orally administered to mice by gavage at a dose of 100 mg/kg of mice body weight. Collect blood, fecal samples, and various major organs at predetermined time points (1, 4, 8, 24 h). After washing and weighing, homogenize the sample, digest the ground sample with concentrated nitric acid for 24 h, and quantitatively determine the content of Co ions using ICP-MS.

Statistical analysis

Statistical data were analyzed using GraphPad Prism7 and SPSS software. The experimental results are represented by mean ± SEM (standard error of the mean). Differences were considered statistically significant when $p<0.05$. **** means $p<0.0001$, *** means $p<0.001$, and ** means $p<0.05$.

Density Functional Theory (DFT) Calculations

In this study, all the calculated results were obtained by using the first-principle method through the Cambridge serial total energy package (CASTEP) module in Materials Studio. The calculations are performed using the density functional theory (DFT) with the Perdew-Burke-Ernzerhof (PBE) functional based on generalized gradient approximation (GGA) and the projector-augmented wave (PAW) method. The cutoff energy of atomic wave functions was set to 400 eV. The k point was set to be 3×3×3. The atom positions were relaxed using the Broyden-Fletcher-Goldfarb-Shanno (BFGS) method. All structures are fully relaxed without any symmetry constraint. The structural optimization parameters are the following: Energy, Max. force and Max. displacement were set to 1.0 e-5 eV/Å 0.03 Ha/Å and 0.001 Å respectively.

Supplementary Figures: Figure S1- S21；Table S1-S5.

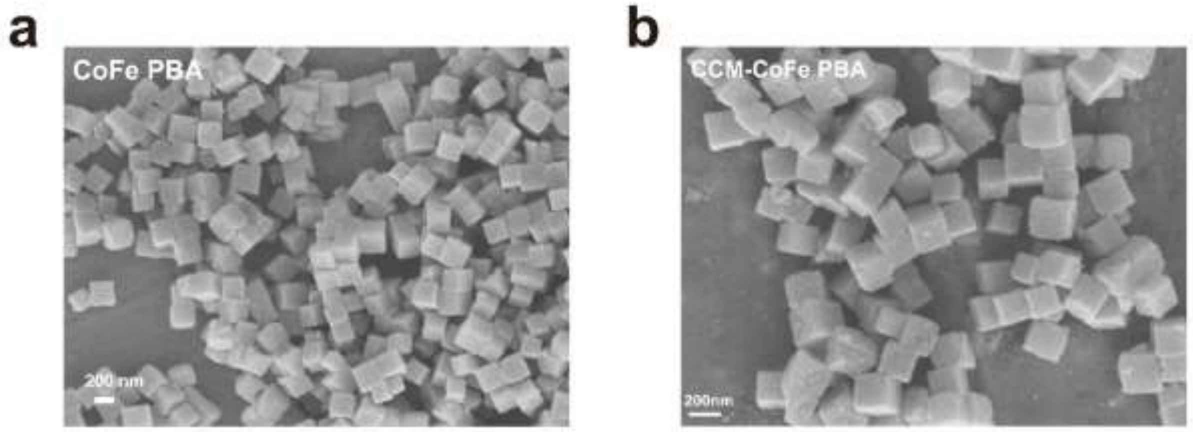

Figure S1. SEM images of a) CoFe PBA and b) CCM-CoFe PBA.

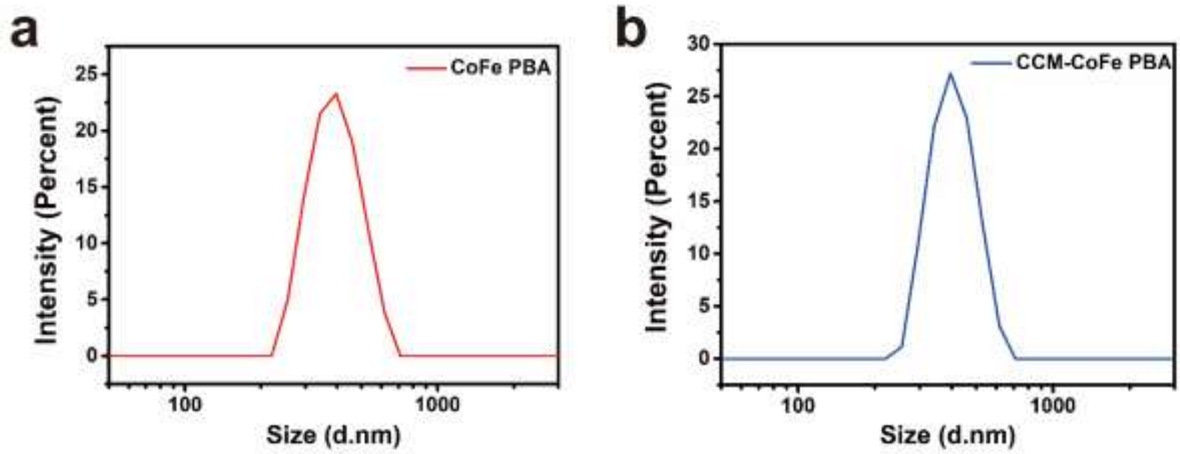

Figure S2. The particle size distribution of a) CoFe PBA and b) CCM-CoFe PBA.

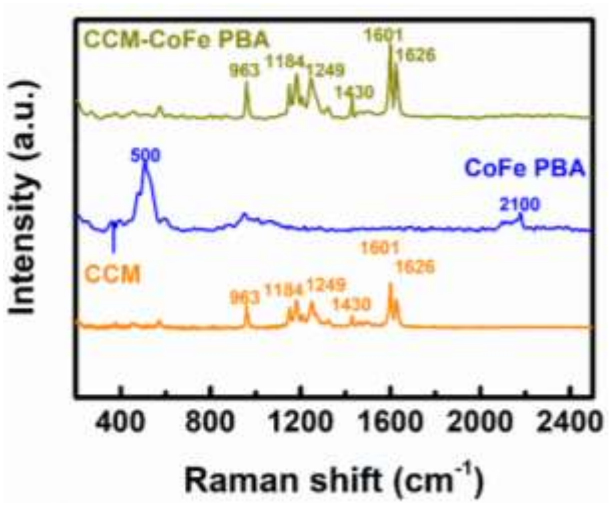

Figure S3. Raman spectra of curcumin, CoFe PBA and CCM-CoFe PBA.

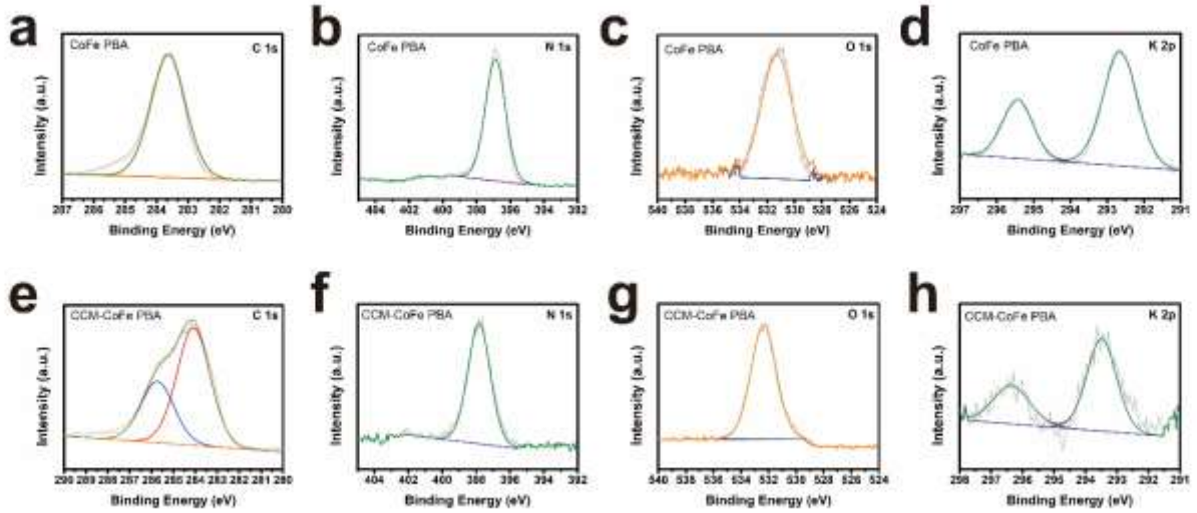

Figure S4. The high-resolution XPS profiles of a) CoFe PBA C 1s, b) CCM-CoFe PBA C 1s, c) CoFe PBA N 1s, d) CCM-CoFe PBA N 1s, e) CoFe PBA O 1s, f) CCM-CoFe PBA O 1s, g) CoFe PBA K 2p and h) CCM-CoFe PBA K 2p.

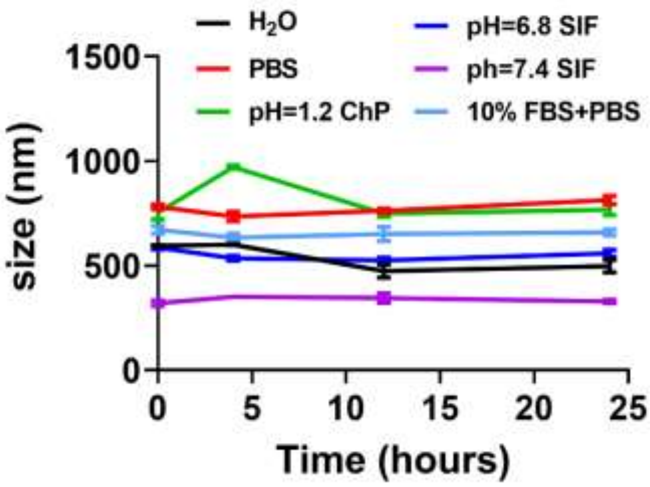

Figure S5. Particle size changes of CCM-CoFe PBA in different biological buffer solutions at different times.

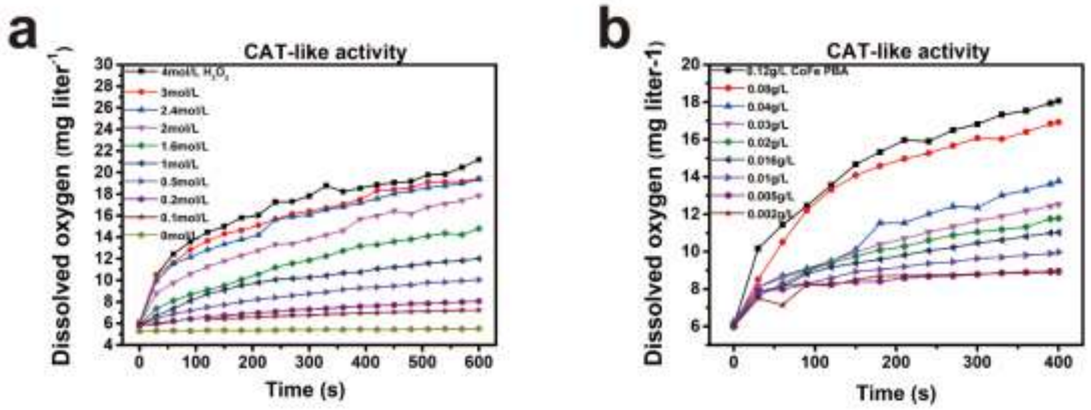

Figure S6. CAT-mimic activity. a) Effects of different concentrations of hydrogen peroxide on H_2O_2 elimination: CoFe PBA (80 μg mL^{-1}), hydrogen peroxide (0.0002 - 4 M), pH7.4. b) Effects of different concentrations of CoFe PBA on H_2O_2 elimination: CoFe PBA (0.002-0.12 mg L^{-1}), hydrogen peroxide (4 mM), pH = 7.4.

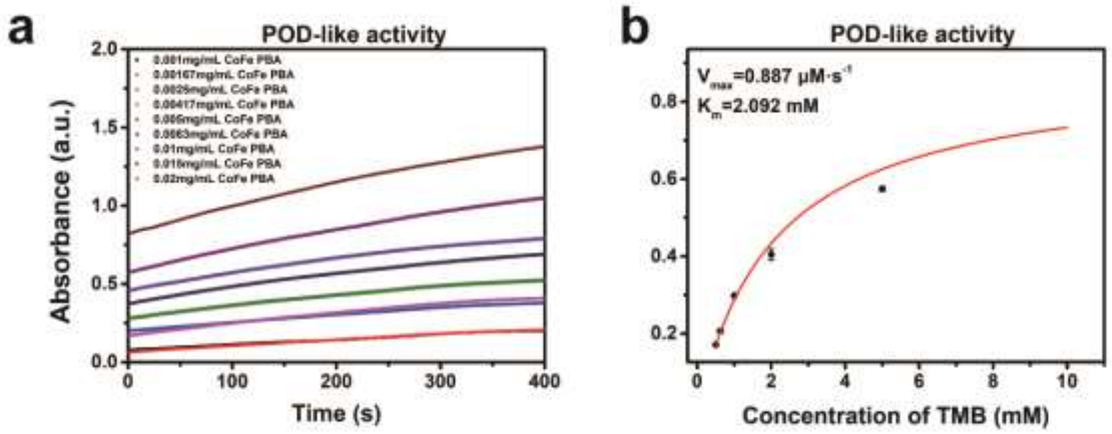

Figure S7. a) Effects of different concentrations of CoFe PBA on peroxide elimination: CoFe PBA (0.001-0.02 mg L^{-1}), hydrogen peroxide (30%, m/m), TMB (0.12 mM), pH = 4.0. b) Steady-state kinetic determination of peroxidase (POD) -like activity of CoFe PBA with different TMB concentrations (6-120 mM).

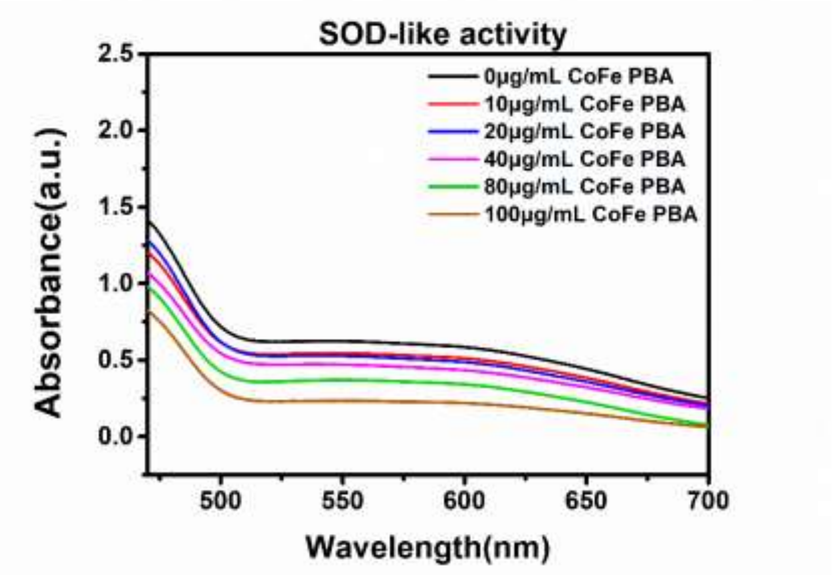

Figure S8. SOD-mimic activity. Different concentrations of CoFe PBA were used to remove superoxide ions, characterized by the absorbance (NBT) of nitrotetrazolium chloride.

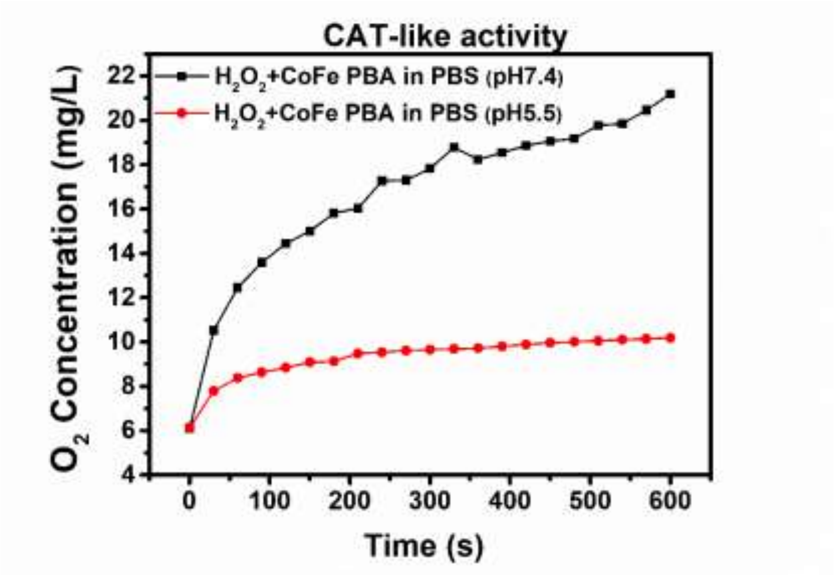

Figure S9. Comparison of CAT-like enzyme activity under different pH conditions.

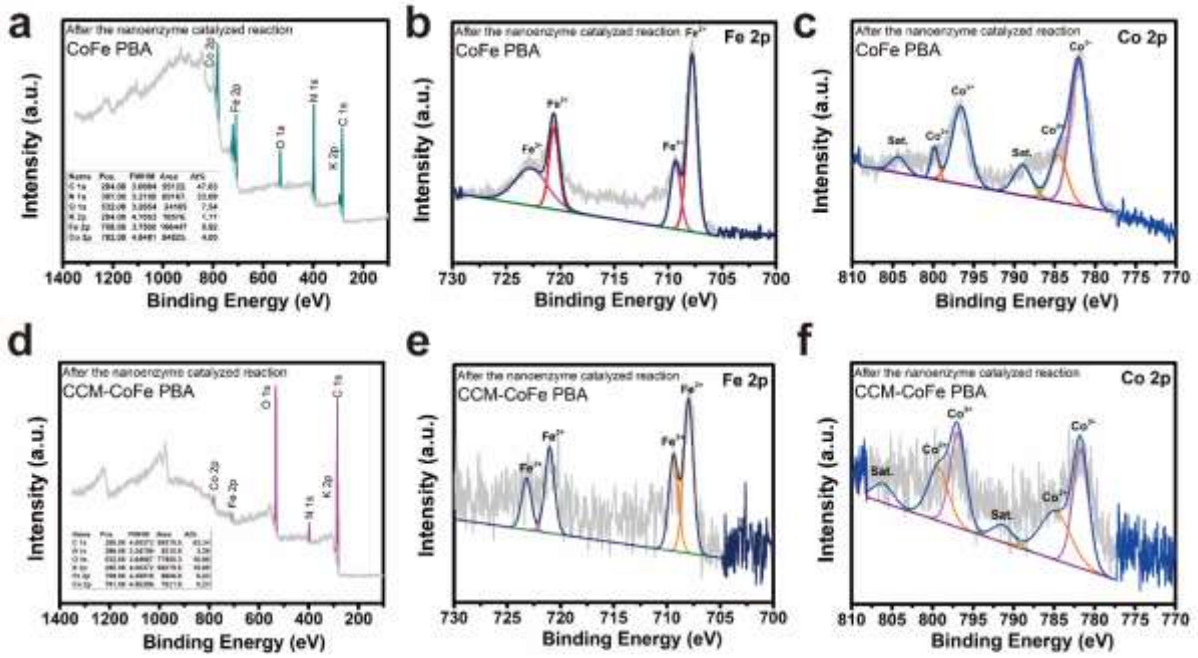

Figure S10. XPS spectrum measurements of CoFe PBA and CCM-CoFe PBA after nano-enzyme test.

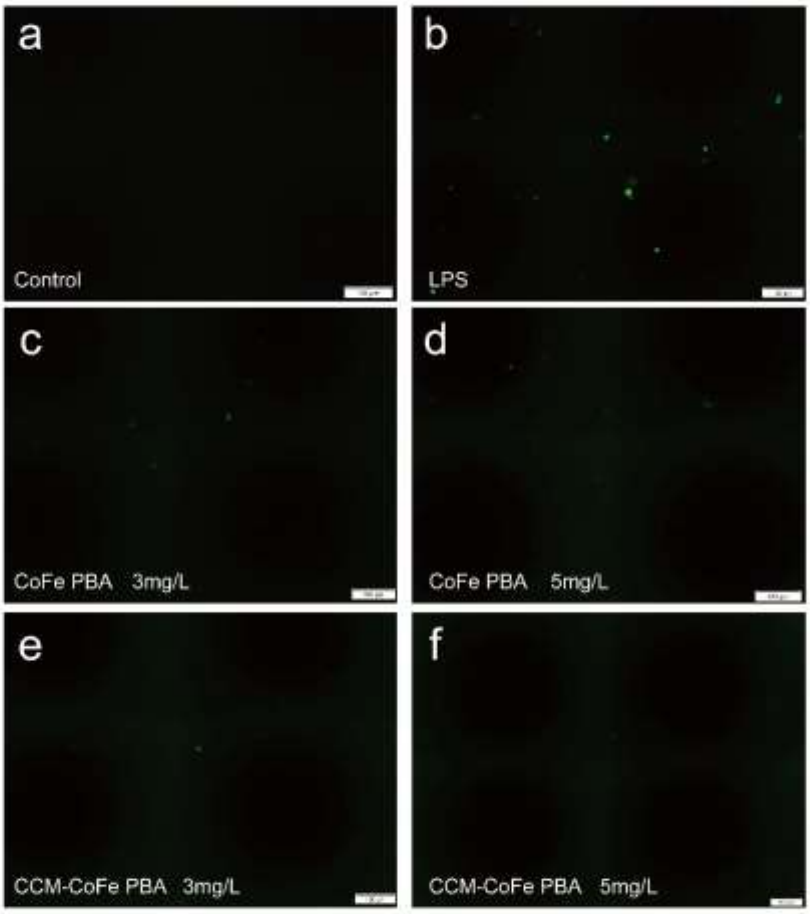

Figure S11. ROS levels in RAW264.7 cells were monitored by laser scanning confocal microscopy in the presence or absence of nano-enzymes. Excitation wavelengths (488 nm), emission wavelengths (525 nm), exposure time 117.647 ms. The scale bar of the DCF field is 100 μm,

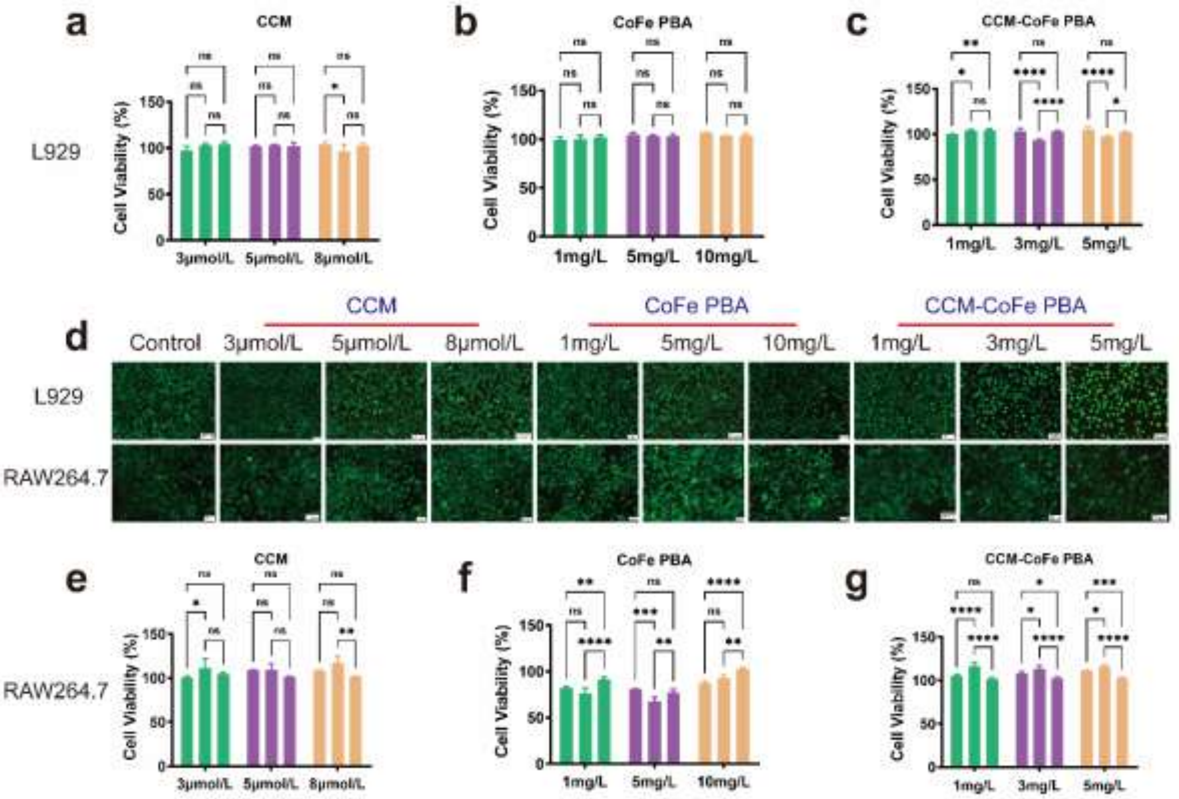

Figure S12. Effects of CCM, CoFe PBA, and CCM-CoFe PBA on the proliferation of L929 and RAW264.7 cells. L929 (a, b, c) and RAW264.7 (e, f, g) cells were co-treated with 3-8 μmol L^{-1} CCM, 1-10 mg L^{-1} CoFe PBA, and 1-5 mg L^{-1} CCM-CoFe PBA for 24 h, 48 h, and 72 h, and CCK-8 analysis was performed. Each value represents the mean ± SD standard deviation of three independent experiments. A combination of calcein acetoxymethyl ester (calcein-AM) and propidium iodide (PI) staining was performed to visualize living and dead cells (d). The scales in d are 50 μm.

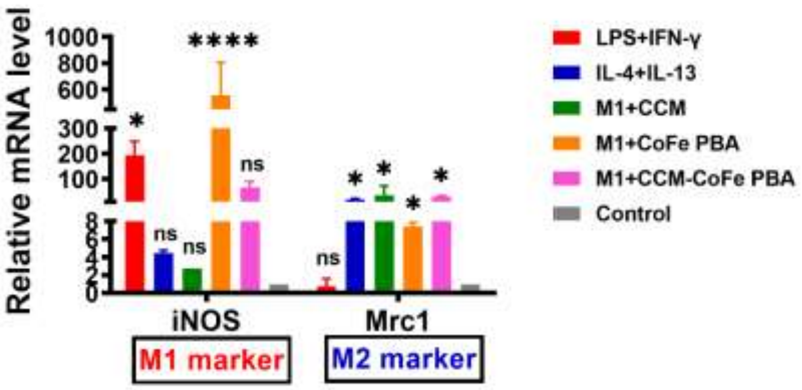

Figure S13. mRNA expression of M1 macrophage marker (iNOS) and M2 macrophage marker (Mrc1) in RAW264.7 was detected by q-PCR under different conditions (n = 3).

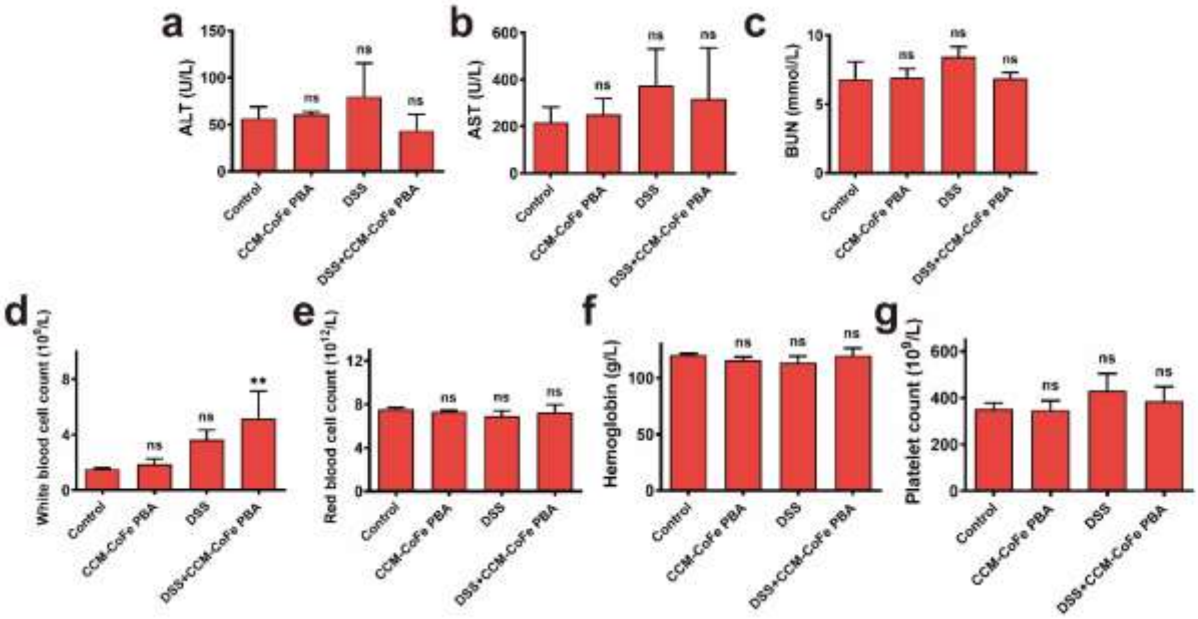

Figure S14. In vivo biocompatibility study. (a-c) Serum biochemical data. (d-g) Blood routine data.

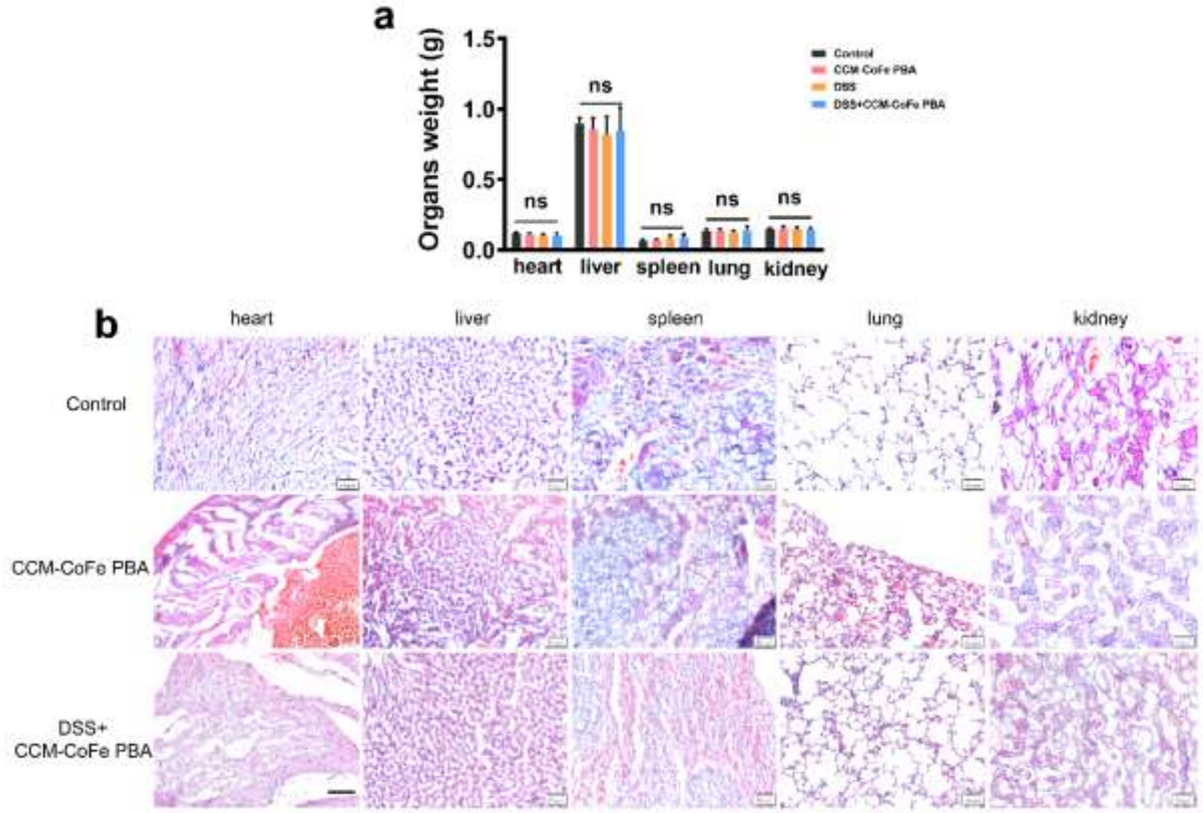

Figure S15. In vivo biocompatibility study. (a) Weights of major organs (heart, liver, spleen, lungs, kidneys) in different groups. (b) Histological sections of major organs (heart, liver, spleen, lung, kidney) from different groups with a scale of 50 µm.

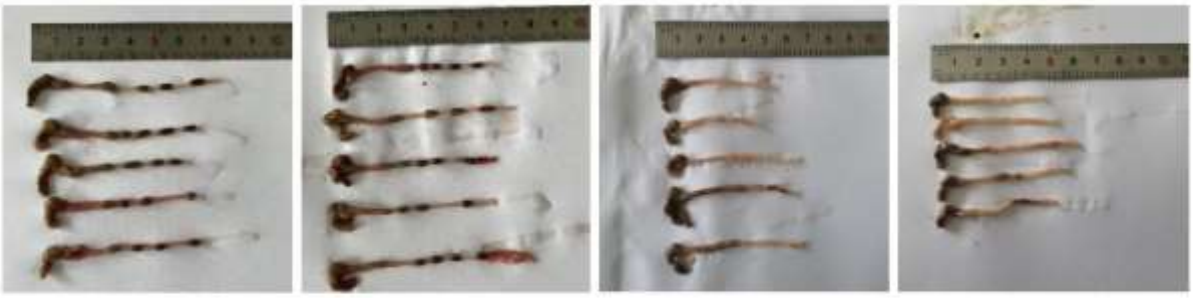

Figure S16. Images of mouse colons on day 8.

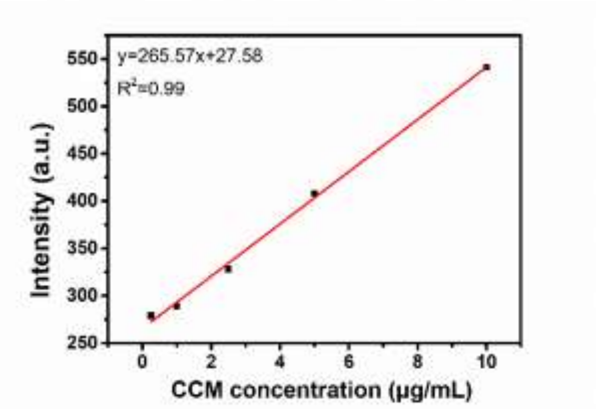

Figure S17. The calibration curve for CCM quantifion by using fluorescent method.

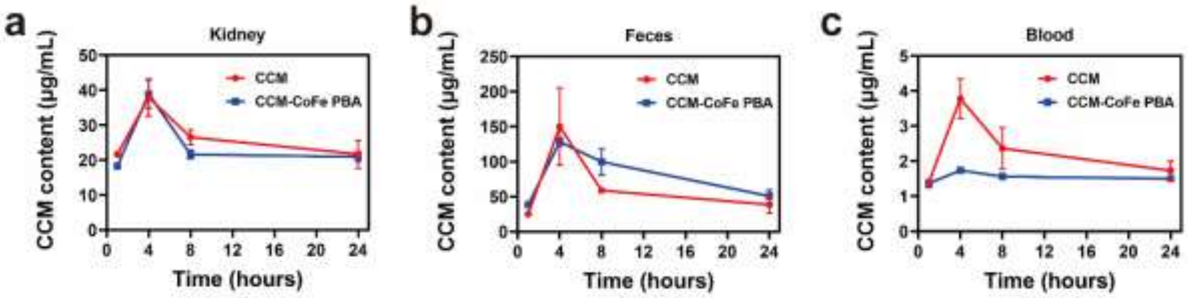

Figure S18. Changes of curcumin concentration and time in kidney, feces and blood after oral administration of free CCM and CCM-CoFe PBA.

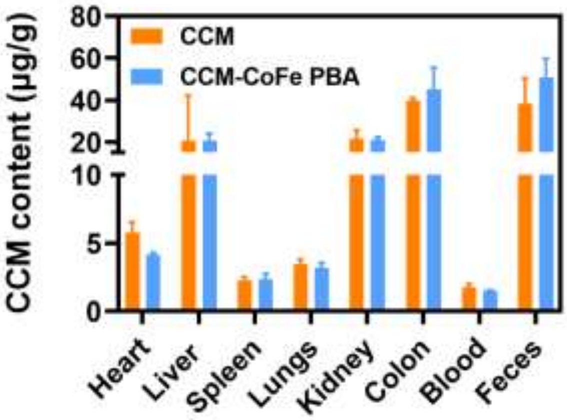

Figure S19. Biodistribution of free CCM and CCM-CoFe PBA after 24 hours of gavage administration.

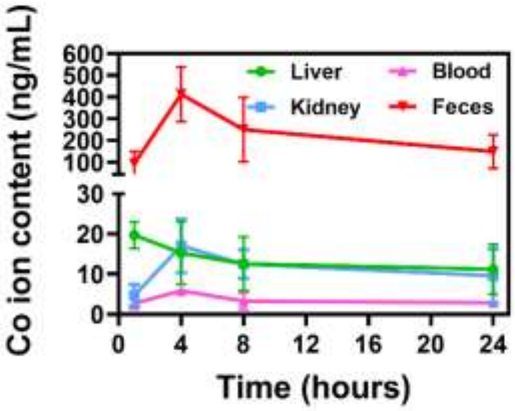

Figure S20. Changes in Co ion concentration and time in mice after intragastric administration of CCM-CoFe PBA.

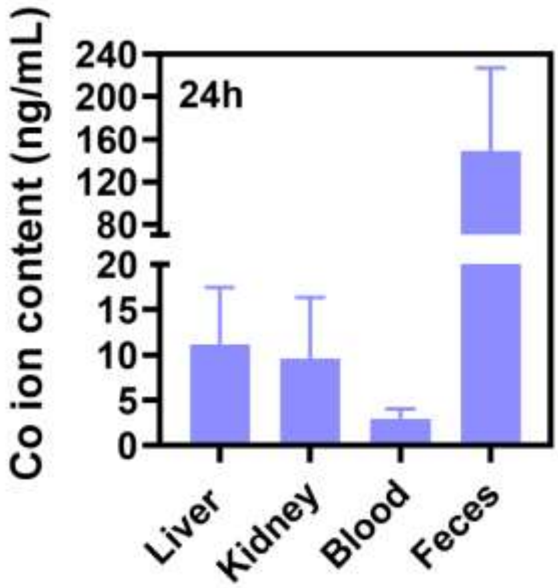

Figure S21. Biodistribution of Co ions after 24 hours of intragastric administration of CCM-CoFe PBA.

Table S1. Zeta potential of a) CoFe PBA and b) CCM-CoFe PBA.

	Zeta Potential Distribution
CoFe PBA	-29.9 mV
CCM-CoFe PBA	-28.6 mV

Table S2. Primer sequences for quantitative q-PCR.

Genes	Forward	Reverse
β-actin	GGTGTGATGGTGGGAATGGG	ACGGTTGGCCTTAGGGTTCAG
CD68	GGTGGAAGAAAGGCTTGGGGCATA	TTCTGCGCCATGAATGTCCACTGT
iNOS	GTTCTCAGCCCAACAATACAAGA	GTGGACGGGTCGATGTCAC
CD163	TCAGCGACTTACAGTTTCCTC	GCCTTTGAATCCATCTCTTG
CD206	CTCTGTTCAGCTATTGGACGC	TGGCACTCCCAAACATAATTTGA
Mrc1	GGCGAGCATCAAGAGTAAAGA	CATAGGTCAGTCCCAACCAAA

Table S3. Feeding schedules for each group.

Groups (number of mice)	Protocol
Control (n=15)	normal saline (NS)
CCM-CoFe PBA (n=15)	normal saline (NS)
DSS (n=15)	3% DSS+ normal saline (NS)
DSS+CCM-CoFe PBA (n=15)	3% DSS+CCM-CoFe PBA (10mg/kg)

Table S4. DAI scores are based on disease marker intensities.

Stool consistency	Bleeding	Weight loss
0=formed	0=normal color	0=no weight loss
1=mild soft	1=brown color stool	1=1%-5% weight loss
2=very soft	2=reddish color stool	2=6%-10% weight loss
3=watery	3=bloody stool	3=11%-15% weight loss
		4=≥16% weight loss

Table S5. Zeta potential changes of CCM-CoFe PBA in different biological buffers at different times.

Time (h)	Zeta potential (mV)					
	H_2O	PBS	pH=1.2 ChP	pH=6.8 SIF	pH=7.4 SIF	10% FBS+PBS
0	-2.88±0.12	-23.13±0.90	6.66±0.79	-14.71±0.80	-15.83±1.81	-9.87±1.17
4	-4.62±0.12	-23.53±0.25	4.40±0.62	-14.58±0.25	-15.82±0.70	-9.25±0.07
12	-5.33±0.58	-24.09±0.75	4.26±0.48	-14.53±0.40	-15.47±1.56	-9.83±0.65
24	-3.53±0.29	-25.13±1.06	4.43±0.49	-13.93±0.58	-16.37±0.50	-9.81±0.51
